# Combining VPS34 inhibitors with STING agonists enhances type I interferon signaling and anti‐tumor efficacy

**DOI:** 10.1002/1878-0261.13619

**Published:** 2024-03-20

**Authors:** Yasmin Yu, Madhumita Bogdan, Muhammad Zaeem Noman, Santiago Parpal, Elisabetta Bartolini, Kris Van Moer, Simone Caroline Kleinendorst, Kristine Bilgrav Saether, Lionel Trésaugues, Camilla Silvander, Johan Lindström, Jodi Simeon, Mary Jane Timson, Hikmat Al‐Hashimi, Bryan D. Smith, Daniel L. Flynn, Andrey Alexeyenko, Jenny Viklund, Martin Andersson, Jessica Martinsson, Katja Pokrovskaja Tamm, Angelo De Milito, Bassam Janji

**Affiliations:** ^1^ Department of Oncology‐Pathology Karolinska Institutet Stockholm Sweden; ^2^ Sprint Bioscience Huddinge Sweden; ^3^ Deciphera Pharmaceuticals Waltham MA USA; ^4^ Tumor Immunotherapy and Microenvironment (TIME) Group, Department of Cancer Research Luxembourg Institute of Health (LIH) Luxembourg; ^5^ Department of Medical Imaging, Nuclear Medicine Radboud University Medical Center Nijmegen The Netherlands; ^6^ Department of Molecular Medicine and Surgery Karolinska Institutet Stockholm Sweden; ^7^ Science for Life Laboratory Solna Sweden; ^8^ Evi‐networks Consulting Huddinge Sweden; ^9^ Department of Microbiology, Tumor and Cell Biology Karolinska Institutet Solna Sweden

**Keywords:** autophagy, cancer, immunotherapy, interferons, STING

## Abstract

An immunosuppressive tumor microenvironment promotes tumor growth and is one of the main factors limiting the response to cancer immunotherapy. We have previously reported that inhibition of vacuolar protein sorting 34 (VPS34), a crucial lipid kinase in the autophagy/endosomal trafficking pathway, decreases tumor growth in several cancer models, increases infiltration of immune cells and sensitizes tumors to anti‐programmed cell death protein 1/programmed cell death 1 ligand 1 therapy by upregulation of C‐C motif chemokine 5 (CCL5) and C‐X‐C motif chemokine 10 (CXCL10) chemokines. The purpose of this study was to investigate the signaling mechanism leading to the VPS34‐dependent chemokine increase. NanoString gene expression analysis was applied to tumors from mice treated with the VPS34 inhibitor SB02024 to identify key pathways involved in the anti‐tumor response. We showed that VPS34 inhibitors increased the secretion of T‐cell‐recruitment chemokines in a cyclic GMP‐AMP synthase (cGAS)/stimulator of interferon genes protein (STING)‐dependent manner in cancer cells. Both pharmacological and small interfering RNA (siRNA)‐mediated VPS34 inhibition increased cGAS/STING‐mediated expression and secretion of CCL5 and CXCL10. The combination of VPS34 inhibitor and STING agonist further induced cytokine release in both human and murine cancer cells as well as monocytic or dendritic innate immune cells. Finally, the VPS34 inhibitor SB02024 sensitized B16‐F10 tumor‐bearing mice to STING agonist treatment and significantly improved mice survival. These results show that VPS34 inhibition augments the cGAS/STING pathway, leading to greater tumor control through immune‐mediated mechanisms. We propose that pharmacological VPS34 inhibition may synergize with emerging therapies targeting the cGAS/STING pathway.

## Graphical abstract

VPS34 inhibition in cancer cells triggers activation of the cGAS–STING pathway and thereby induces a type I interferon response leading to CCL5/CXCL10 chemokine secretion. VPS34 inhibitor treatment in combination with STING agonists sensitizes the STING‐dependent proinflammatory cytokine response. VPS34 inhibitor SB02024 in combination with STING agonist ADU‐S100 treatment decreases tumor growth and increases survival of tumor‐bearing mice.

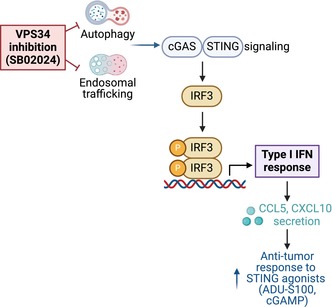

**Abbreviations**
ADME: absorption, distribution, metabolism, and excretion
ATG5: autophagy‐related 5
CCL5: C‐C motif chemokine 5
cGAMP: cyclic GMP‐AMP
cGAS: cyclic GMP‐AMP synthase
CRC: colorectal cancer
CXCL10: C‐X‐C motif chemokine 10
CYP: cytochrome P450
ELISA: enzyme‐linked immunosorbent assay
FaSSIF: fasted state simulated intestinal fluid
FDR: false discovery rate
FRET: fluorescence resonance energy transfer
FYVE domain: Fab1, YOTB, Vac1, and EEA1 domain
Hep: hepatocyte
ICB: immune checkpoint blockade
IFN: interferon
IO: immuno‐oncology
IRF: interferon regulatory factor
ISRE: interferon‐stimulated response element
JAK: janus kinase
Log*D*
: distribution coefficient
MAPK: mitogen‐activated protein kinase
MOA: mechanism of action
MSD: mesoscale discovery
NEA: network enrichment analysis
NFκB: nuclear factor kappa‐light‐chain‐enhancer of activated B cells
NK cell: natural killer cell
Papp: apparent permeability coefficient
PD: pharmacodynamic
PD‐1: programmed cell death protein 1
PDB: protein data bank
PD‐L1: programmed cell death 1 ligand 1
PI : PS: ratio of phosphatidylinositol (PI) to phosphoserine (PS)
PI3K: phosphoinositide 3‐kinase
PI3P: phosphatidylinositol‐3‐phosphate
PK: pharmacokinetic
PPB: plasma protein binding
PXR: pregnane X receptor
RCC: renal cell carcinoma
RT‐PCR: reverse transcription polymerase chain reaction
SEAP: secreted embryonic alkaline phosphatase
SGF: simulated gastric fluid
siRNA: small interfering RNA
STAT: signal transducer and activator of transcription
STING: stimulator of interferon genes protein
TGF: transforming growth factor
TME: tumor microenvironment
Vd: volume of distribution
VPS34: vacuolar protein sorting 34
VPS34i: VPS34 inhibitor

## 1 Introduction

Macroautophagy (hereafter autophagy) is a cellular maintenance process enabling the recycling of cytoplasmic components via the formation of double‐membraned vesicles termed autophagosomes. Upon fusion with lysosomes, the cargo of the autophagosome is degraded and released back into the cytosol fueling cellular metabolism. Due to its cytoprotective role, autophagy has been implicated in several human pathologies including cancer [1]. The role of autophagy in cancer is complex and highly context dependent. Cancer cells induce autophagy in response to stress factors present in the tumor microenvironment (TME) such as hypoxia and nutrient deprivation, as well as a resistance mechanism to cancer therapy [2]. Recent reports have pointed out a key role of autophagy in tumor immune evasion by decreasing the release of inflammatory mediators [3, 4, 5], reducing immune cell recognition [6], and limiting T and natural killer (NK) cell‐mediated cell death [7, 8, 9, 10]. In addition, autophagy was shown to suppress anti‐tumor T cell responses [5, 11]. This has revived interest in targeting autophagy pharmacologically to attenuate immune evasion and as a potential combination strategy to improve cancer immunotherapy.

Efforts to develop autophagy inhibitors have largely been focused on targeting early steps of autophagy, with the lipid kinase vacuolar protein sorting 34 (VPS34) as a promising target [12, 13, 14, 15, 16]. VPS34, also referred to as phosphatidylinositol 3‐kinase catalytic subunit type 3 (PIK3C3), regulates autophagy initiation among other vesicular trafficking processes [17]. We recently reported that both genetic and pharmacological VPS34 inhibition leads to increased effector immune cell infiltration and synergizes with PD‐1/PD‐L1 blockade therapy in murine melanoma and colorectal cancer (CRC) models [18].

Here, we investigate the mechanisms underlying the improvement of the anti‐tumor immune response following VPS34 inhibitor (VPS34i) treatment. Consistent with our observations in melanoma and CRC models, VPS34 inhibition in a renal cancer model results in an inflammatory signature in the tumor. Mechanistically, we show that VPS34 inhibition triggers an interferon (IFN) response dependent on the cGAS‐STING pathway in renal and melanoma cancer cells. We further demonstrate that VPS34i treatment synergizes with STING agonists in increasing a STING‐dependent IFN response *in vitro*. Also, combination treatment with a STING agonist and VPS34i significantly decreases tumor growth and prolongs mice survival in tumor‐bearing mice compared to single agent administration of the STING agonist or VPS34 inhibitor. These findings identify pharmacological VPS34 inhibition as a therapeutic strategy to activate a proinflammatory IFN response in cancer cells which could be beneficial to improve the outcome of cancer immunotherapy.

## 2 Materials and methods

### 2.1 Cell lines and reagents

Human embryonic kidney 293T (RRID:CVCL_0063, #CRL‐3216), human non‐small‐cell lung cancer (NSCLC) H1299 (RRID:CVCL_0060, #CRL‐5803), human RCC 786‐O (RRID:CVCL_1051, #CRL‐1932), murine melanoma B16‐F10 (RRID:CVCL_0159, #CRL‐6475), murine melanoma YUMM1.7 (RRID:CVCL_JK16, #CRL‐3362), murine colon carcinoma CT26 (RRID:CVCL_7256, #CRL‐2638), and murine breast cancer 4T1 (RRID:CVCL_0125, #CRL‐2539) cells were all purchased from ATCC (Manassas, VA, USA). Murine RCC Renca (RRID:CVCL_2174, #440321) cells were purchased from CLS (Eppelheim, Germany). Human RCC A‐498 (RRID:CVCL_1056, #ACC 55) cells were purchased from DSMZ (Braunschweig, Germany). H1299‐GFP‐2xFYVE cells were generated and cultured as previously described [16]. Human melanoma Me30966 cells were derived from the Istituto Nazionale dei Tumori, Milan, Italy [19]. Human THP‐1‐Dual™ reporter monocytes (RRID:CVCL_X599, STING present #thpd‐nfis, STING‐knockout #thpd‐kostg) purchased from InvivoGen (San Diego, CA, USA) and murine DC2.4 dendritic cells (RRID:CVCL_J409, # SCC142) purchased from Sigma‐Aldrich (St. Louis, MO, USA) were cultured according to supplier's instructions. 293T cells were cultured in DMEM (#11995066); Renca, CT26, 4T1, and Me30966 cells were cultured in RPMI‐1640 (#21875091); 786‐O cells were cultured in RPMI‐1640 (ATCC modification, #A1049101); A‐498 cells were cultured in MEM (#31095029), B16‐F10 and YUMM1.7 cells were cultured in DMEM/F12 (#11330057). All culture media were purchased from Thermo Fisher (Waltham, MA, USA) and supplemented with 10% fetal bovine serum (#35‐079‐CV; Corning Inc., Corning, NY, USA; or #SH30010.03; HyClone, Chicago, IL, USA). All cells were maintained at 37 °C under 5% CO_2_ and routinely tested for mycoplasma contamination using the MycoAlert kit (#LT07‐318; Lonza, Basel, Switzerland). Murine cell lines B16‐F10, YUMM1.7, CT26, and 4T1 have been authenticated by Microsynth (Balgach, Switzerland) using highly polymorphic short tandem repeat loci (STRs) as published in Almeida et al. [20]. Fragment analysis was conducted on an ABI 3730xl instrument (Thermo Fisher), and the resulting data were analyzed using genemarker software (Softgenetics, State College, PA, USA). All experiments were performed with cells cultured for < 15 passages to ensure consistency and reproducibility of experimental results.

The VPS34 inhibitor SB02024 was provided by Sprint Bioscience (Huddinge, Sweden). The VPS34 inhibitor SAR405 (#S7682; Selleckchem, Houston, TX, USA), the STING agonist ADU‐S100 (#CT‐ADUS100; ChemieTek, Indianapolis, IN, USA; or #HY‐12885; MedChemExpress, Monmouth Junction, NJ, USA), and 2′,3′‐cGAMP (cGAMP) (#TLRL‐NACGA‐1; InvivoGen) were used according to manufacturer's instructions.

### 2.2 VPS34 protein production

VPS34 expression vector (pNIC28‐Bsa4) coding for protein corresponding to amino acid sequence S282‐H879 of human VPS34 (VPS34ΔC2) was obtained from the SGC clone collection (Source Bioscience, Nottingham, UK). BL21(DE3)‐R3‐pRARE2 cells transformed with the VPS34ΔC2 expression vector were cultivated in TB supplemented with Kanamycin (50 μL·mL^−1^), Chloramphenicol (34 μg·mL^−1^) and glycerol (8 g·L^−1^) at 37 °C until OD600 = 1.6. Then, the temperature was lowered to 18 °C and expression induced by the addition of 0.5 mm IPTG. Cells were harvested by centrifugation after 20 h. The pellet was resuspended in lysis buffer (20 mm HEPES pH 7.5, 500 mm sodium chloride, 10% glycerol, 10 mm imidazole, 0.5 mm TCEP) and homogenized in an emulsiflex homogenizer. Ni‐sepharose FF matrix was added to the crude lysate and batch binding was performed for 1.5 h at 4 °C. The matrix was separated from the unabsorbed lysate by centrifugation and was packed in a XK‐26 column. The column was connected to an ÄKTA FPLC system and washed with IMAC lysis buffer followed by IMAC wash buffer (20 mm HEPES pH 7.5, 500 mm sodium chloride, 10% glycerol, 25 mm imidazole, 0.5 mm TCEP). The bound protein was eluted in 100% IMAC elution buffer (20 mm HEPES pH 7.5, 500 mm NaCl, 10% glycerol, 500 mm imidazole, 0.5 mm TCEP). The protein eluted from the IMAC column was further purified by gel filtration chromatography on a HiLoad Superdex 200 26/60 column equilibrated in Gel filtration buffer (20 mm HEPES pH 7.5, 300 mm sodium chloride, 10% glycerol, 0.5 mm TCEP). His‐tag was removed by incubating VPS34ΔC2 (4.3 mg·mL^−1^) with His‐tagged TEV protease (final concentration 0.05 mg·mL^−1^) overnight at 4 °C. A reversed IMAC purification step was performed to remove TEV protease and His‐tag from the cleaved protein sample. The protein was concentrated, and buffer was exchanged to formulation buffer (20 mm Tris pH 7.5, 300 mm sodium chloride, 10% glycerol, 0.5 mm TCEP) by diafiltration in a Vivaspin 20 device with 10 kDa MWCO (Sartorius, Göttingen, Germany).

### 2.3 Crystallization of complexes between human VPS34 and SB02024

VPS34ΔC2 (30 mg·mL^−1^) was incubated with 1 mm SB02024 for 30 min on ice. The sample was centrifuged for 30 min at 20 000 ***g*** (4 °C). The supernatant was used in crystallization trials. Crystals were obtained in sitting drops using the vapor diffusion technique in a 96‐well plate. 0.15 μL of VPS34ΔC2/SB02024 complex was mixed with 0.15 μL of reservoir solution composed of 22.5% PEG3350, 0.2 m ammonium acetate, and 0.1 m HEPES pH 8.0. Drops were equilibrated at 4 °C against 50 μL of reservoir solution. Plates‐shaped crystals appeared within a week. One microliter of cryoprotective solution (15% PEG3350, 0.15 m ammonium acetate, 0.2 m sodium chloride, 0.1 m HEPES pH 8.0, 1% DMSO, and 25% glycerol) was added on top of the crystallization drop. After 30 s, crystals were harvested and flash‐frozen into liquid nitrogen. Data collection, structure determination, and refinement statistics are described in Table S1. The structure of VPS34ΔC2/SB02024 complex and merged data was deposited in the PDB under accession code 8RXR.

### 2.4 VPS34 biochemical and NanoBRET binding assay

Compound dilution series was prepared in DMSO and further diluted to 4× final assay concentration in assay buffer (Buffer Q #PV5125, Thermo Fisher, supplemented with 2 mm DTT and 2 mm MnCl_2_). 2.5 μL of the diluted compounds were added to a 384‐well assay plate followed by 2.5 μL of 16.5 nm VPS34 enzyme (#PV5126; Thermo Fisher) and incubated for 15 min. Five microliters of substrate mix (20 μm ATP #PV3227 and 200 μm PI : PS substrate #PV5122; Thermo Fisher) in assay buffer was added, mixed, and incubated for 1 h. Five microliters of stop‐detection mix (Adapta kinase assay #PV5099; Thermo Fisher) were added, mixed, and incubated for 30 min and read with Artemis microplate reader. Percent remaining activity as compared to DMSO‐treated control samples was calculated and IC_50_ values were generated using graphpad prism (GraphPad Software, Inc., San Diego, CA, USA). The NanoBRET assay was performed using the manufacturer's protocol. Briefly, 293T cells were transfected with the Kinase‐NanoLuc® Fusion Vector DNA overnight. The next day cells were treated with compound and the NanoBRET tracer reagent and then read out measuring donor and acceptor emission wavelength (450 and 610 nm).

### 2.5 Assessment of ADME properties

Experiments were conducted at Aragen Life Sciences Ltd. (Hyderabad, India) according to internal standard procedures.

### 2.6 Pharmacokinetic and pharmacodynamic profiling of SB02024

The study was performed by Truly Translational AB (Lund, Sweden) following Swedish legislations and approved by the ethical committee Malmö/Lunds djurförsöksetiska nämnd (M120‐15). Animals were housed 5/cage in polysulfone IVC cages at a constant temperature with 12 h light‐dark cycles and free access to food, tap water, and cage enrichment. Animals were allowed to acclimatize to the housing conditions for at least a week before the start of experiments. Female 8‐week‐old NOD.Cg‐Prkdc^*scid*^ Il2rg^*tm1Sug*^/JicTac (CIEA‐NOG®) mice (supplied by Taconic Biosciences, Rensselaer, NY, USA; *n* = 42) were implanted subcutaneously with 1.75 × 10^6^ H1299‐GFP2xFYVE cells in serum‐free RPMI in 50% Matrigel. When tumors reached 300–400 mm^3^ mice were randomized and dosed with 9 or 29 mg·kg^−1^ SB02024 or vehicle (PEG 200 (98%)/Polysorbate (2%)). At indicated time points after dosing, blood samples were taken by heart puncture and tumors were removed. Pharmacokinetic (PK) analysis (plasma and tumor samples) was performed by Q&Q Labs (Gothenburg, Sweden). Plasma was precipitated with acetonitrile, and after centrifugation, the soluble material was recovered. Tumor of 50–150 mg was homogenized mechanically in the presence of 5× acetonitrile relative to tissue weight using Precellys Tissue Homogenizer (Bertin Technologies, Montigny‐le‐Bretonneux, France). Samples were centrifuged and soluble material was kept for further analysis. SB02024 levels were determined using UHPLC‐QQQ: Agilent 6460 (LC–MS/MS; Agilent Technologies, Santa Clara, CA, USA). Pharmacodynamic (PD) analysis of GFP2xFYVE punctae was performed by Offspring Biosciences (Södertalje, Sweden). H1299‐GFP2xFYVE tumors were fixed by immersion in 4% formalin in PBS for 72 h at 4 °C, sampled randomly by six biopsies (diameter 1 mm), and reassembled into three separate tumor microarrays (TMAs). Each TMA block was sectioned and analyzed at eight equally spaced levels. The immunohistochemical staining was performed using the Ventana Discovery XT protocol. GFP antibody (#ab183734; Abcam, Cambridge, UK) was diluted in Ventana Dilution Buffer (#ADB250; Ventana Medical Systems, Oro Valley, AZ, USA). All sections were scanned using a digital whole slide scanner (Pannoramic 250 Flash I; 3D Histech, Budapest, Hungary). Data are represented as the area of the GFP‐2xFYVE positive signal in punctae versus the area of the GFP‐2xFYVE signal diffuse.

### 2.7 Tumor growth inhibition studies

Study protocols were approved by the Institutional Animal Care and Use Committee of CrownBio in China (AN‐1903‐05‐313) or the LIH's animal welfare committee in Luxembourg (agreement LECR‐2018‐12). Mice were handled according to European Union guidelines, adhering to the principles of replacement, reduction, and refinement (3Rs). Animals were housed in appropriate accommodations conducive to their well‐being, with provisions for housing, feeding, and environmental enrichment to meet their physical and behavioral needs. Highly skilled personnel were involved in carrying out procedures related to the animal experiments to ensure proper care and handling. Female 6 to 8‐week‐old BALB/c (supplied by Shanghai Lingchang Biotechnology Co., Shanghai, China; *n* = 14) or C57BL/6 mice (supplied by Janvier Labs, Le Genest‐Saint‐Isle, France; *n* = 40) were injected into the right flank with 1 × 10^6^ Renca or 0.2 × 10^6^ B16‐F10 cells, respectively, resuspended in 100 μL PBS (day 0). When tumors were palpable (day 9 for Renca, day 7 for B16‐F10 tumors), mice were randomly assigned into two groups for the Renca model with *n* = 6 (vehicle) and *n* = 8 (SB02024) or four groups for the B16‐F10 model with *n* = 10. Daily treatment with 20 mg·kg^−1^ SB02024 or vehicle (0.5% methylcellulose in water supplemented with 1% Tween‐80) was administered by oral gavage (day 9–16 for Renca, day 7–16 for B16‐F10 model). For combination studies, ADU‐S100 (10 μg in 40–50 μL PBS) or vehicle (PBS) treatment was administered by intratumoral injection (days 9, 11, 13, and 15). Tumor growth was measured using a caliper every other day and estimated as follows: volume (cm^3^) = (width)^2^ × length × 0.5.

### 2.8 mRNA gene expression analysis

Renca tumor RNA was isolated using the Rneasy Plus kit (#74134; Qiagen, Venlo, the Netherlands), quantified using a NanoDrop spectrophotometer (Thermo Fisher), and 100 ng was used as input for the nCounter PanCancer IO 360 mouse panel (NanoString Technologies, Seattle, WA, USA). Data were analyzed by NanoString's data analysis service. Network enrichment analysis (NEA) was carried out by Evi‐networks, Sweden (see detailed description in Table S2).

### 2.9 Transfection with siRNA

Cells were plated and simultaneously reserve‐transfected using Lipofectamine™ RNAiMAX (#13778030; Thermo Fisher) for 48 h before drug treatment for 24 h. For tandem‐transfections using two separate siRNAs, cells were reverse‐transfected for 24 h followed by forward transfection for 48 h. Cells were transfected with 10 nm non‐targeting pool si*Scramble* (si*SCR*) or 10 nm targeting siRNA (see Table S3).

### 2.10 Western blotting

Cells were washed in PBS and lysed in RIPA buffer (50 mm Tris HCl pH 7.4, 150 mm NaCl, 1% Triton X‐100, 0.5% Na‐deoxycholate, 0.1% SDS) containing protease inhibitor cocktail (#11836170001; Roche, Basel, Switzerland), and phosphatase inhibitor (#4906845001; Roche) for 10 min at 4 °C and centrifuged at 20 000 ***g*** for 10 min at 4 °C. The supernatant was obtained and protein concentration was quantified using BCA assay (#23225; Thermo Fisher). Equal protein amounts with 0.1 m DTT and LDS Sample Buffer (#84788; Thermo Fisher) were heated for 5 min at 90 °C, loaded onto 4–12% Bis‐Tris gels followed by transfer to nitrocellulose membranes. Membranes were blocked in 5% non‐fat dry milk in TBS with 0.05% Tween (TBS‐T) for 1 h, washed in TBS‐T, and probed with primary antibodies (see Table S3) overnight at 4 °C. After washing, membranes were incubated with secondary antibodies (see Table S3) for 1 h. Protein bands were visualized using the Odyssey CLx imaging system (LI‐COR, Lincoln, NE, USA). Images were processed using image studio lite software version 5.2.5 (LI‐COR). Same‐sized rectangular regions of interest (ROIs) were defined around bands of interest and housekeeping gene bands and background correction was applied. Band intensities were quantified using the software, and the intensity of target protein bands was normalized to the corresponding housekeeping gene bands (e.g., Actin). For phospho‐proteins, normalization was performed to total protein levels and housekeeping controls. Final normalization was conducted to experimental controls.

### 2.11 Quantitative RT‐PCR

RNA was isolated using the PureLink kit (#12183018A; Thermo Fisher) and DNA‐binding columns (Rneasy plus kit, #74134; Qiagen) and/or Dnase treatment (#12185010; Thermo Fisher). After quantification using NanoDrop, 50 ng·μL^−1^ RNA was used for cDNA synthesis with SuperScript IV VILO Master Mix (#11756050; Thermo Fisher). Quantitative RT‐PCR was run with diluted cDNA (1 : 25 or 1 : 12.5), PowerUp SYBR Green Master Mix (#A25741; Thermo Fisher), and 200 nm primers (see Table S3) on a CFX Connect RT‐PCR system (BioRad, Hercules, CA, USA). Data were analyzed using the ΔΔCT method.

### 2.12 Cytokine assays

Cell culture medium was centrifuged at 300 ***g*** for 10 min at 4 °C and supernatant stored at −80 °C. Mouse blood was collected at termination in K2‐EDTA tubes on ice, centrifuged at 1270 ***g*** for 10 min at 4 °C, and plasma was stored at −80 °C. Cytokine levels were quantified using Mesoscale Discovery (MSD) assays: human CCL5 (#F21ZN), CXCL10 (#K151UFK), IFNβ (#K151VIK); mouse CCL5 (#K152A2K) and CXCL10 (#K152UFK). Low concentrations of human IFNβ were determined using High‐Sensitivity IFNβ ELISA (#41415‐1; PBL Assay Science, Piscataway, NJ, USA). CCL5/CXCL10 levels in some experiments were quantified using R&D Systems (Minneapolis, MN, USA) ELISAs: human CCL5 (#DY278) or CXCL10 (#DY266); mouse CCL5 (#DY478).

### 2.13 THP‐1‐dual reporter assay

Reporter assays were performed according to manufacturer's protocols (InvivoGen). Briefly, 1.5 × 10^5^ cells were plated in 96‐well plates and 20 μL of medium supernatants were analyzed after overnight treatment.

### 2.14 Melanoma patient data mining

Data from the TCGA skin cutaneous melanoma (SKCM) cohort (448 patients) were downloaded from cBioPortal (http://www.cbioportal.org/). IDs of patients displaying high and low *cGAS* mRNA expression (*z*‐score relative to all samples) were extracted. Each patient's vital status and survival values (overall survival, OS; disease‐specific survival, DSS) were downloaded from the TCGA database. In patients displaying high and low *cGAS*, the log_2_ mRNA expression levels (batch normalized from Illumina HiSeq_RNASeqV2) of *CCL5*, *CXCL10*, markers for CD8 (*CD8A*, *CD8B*) and markers for NK (*NCR1*, *NCR3*) cells were retrieved. The differential expression of genes of interest was assessed using graphpad software. The median survival and *P*‐value were calculated using the log‐rank (Mantel‐Cox) test in graphpad prism software. The OS and DSS information of the melanoma patients expressing high and low *cGAS* are reported in Tables S4 and S5, respectively. The expression values of *CCL5*, *CXCL10*, *CD8A*, *CD8B*, *NCR1*, and *NCR3* are reported in Table S6.

### 2.15 Statistical analysis

All statistical analyses except for NEA scores were performed using graphpad prism software version 9.0.2. When comparing the means of two groups an unpaired two‐tailed Student's *t*‐test was performed, whereas when comparing more than two groups, a one‐way ANOVA was performed followed by multiple comparison tests as indicated. *P*‐values of < 0.05 were considered statistically significant. Differential pathway activation (NEA scores) was evaluated in an R environment using standard functions anova and lm from package stats. A false discovery rate (FDR) < 0.1 was used as a cut‐off for statistical significance for NEA.

## 3 Results

### 3.1 Co‐crystallization of VPS34 and SB02024 reveals the molecular basis for potency and kinome selectivity

**Fig. 1:**
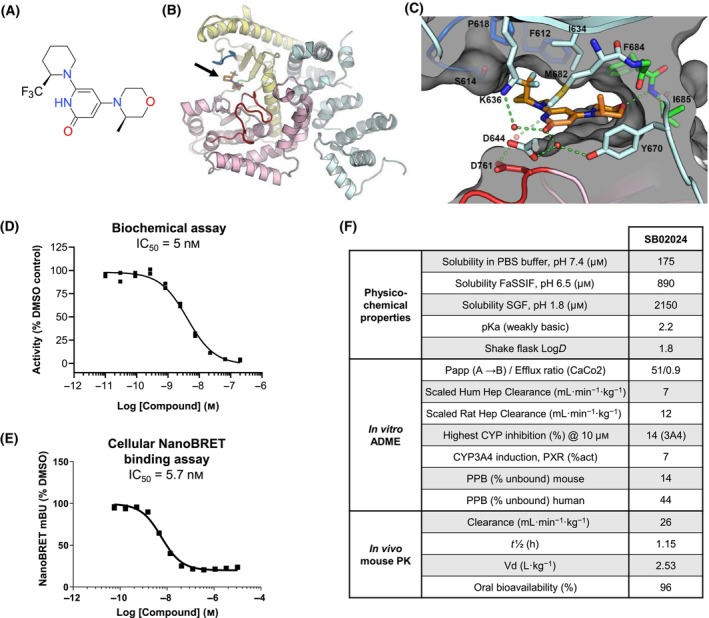
Identification of SB02024 as a potent and selective inhibitor of VPS34. (A) Chemical structure of SB02024. (B) Cartoon representation of the structure of complex between VPS34ΔC2 and SB02024. The N‐, C‐lobes, and helical domains of VPS34ΔC2 are colored in yellow, pink, and light cyan, respectively. The hinge region, P‐loop, and activation loop are colored in green, blue, and red, respectively. SB02024 is displayed as sticks with its carbon atoms colored orange. SB02024 location in VPS34ΔC2 active site is pinpointed by a black arrow. (C) SB02024 binding‐site with color code as in panel B. SB02024, protein sidechain, and main‐chain atoms involved in interactions with SB02024 are displayed as sticks. Water molecules interacting with SB02024 are shown as red spheres. Hydrogen bonds are represented by green dashes. The molecular surface and a slice through the molecular surface are colored in gray. The structures in panels B and C were generated using pymol (The PyMOL Molecular Graphics System, Version 2.5; Schrödinger, LLC, New York, NY, USA). (D) Potency of SB02024 in a biochemical assay. VPS34 enzyme was incubated with different concentrations of SB02024 and IC_50_ was determined. The dose–response curve shows an average of two representative experiments of *n* = 12 independent experiments. (E) Potency of SB02024 in NanoBRET assay. HEK293T cells transfected with VPS34 incubated with different concentrations of SB02024 and IC_50_ were determined. The dose–response curve shows a representative experiment of *n* = 4 independent experiments. mBU, milliBRET units. (F) Key SB02024 physiochemical and absorption, distribution, metabolism, and excretion (ADME) properties. CYP, cytochrome P450; FaSSIF, fasted state simulated intestinal fluid; Hep, hepatocyte; Log*D*, distribution coefficient; Papp, apparent permeability coefficient; PK, pharmacokinetics; PPB, plasma protein binding; PXR, pregnane X receptor; SGF, simulated gastric fluid; Vd, volume of distribution.

We have previously characterized SB02024, designed using a fragment‐based drug discovery approach, as a potent and selective inhibitor of VPS34 [16]. In this report, we describe for the first time the binding mode of SB02024, a 2‐pyridone substituted in the 4‐position by (R)‐3‐methylmorpholine and in the 6‐position by (R)‐2‐(trifluoromethyl)piperidine (Fig. 1A), to the active site of VPS34, and its drug‐like properties. To determine the binding mode, SB02024 was co‐crystallized with a VPS34 protein construct lacking the C2 N‐terminal domain hereafter referred to as VPS34ΔC2. The complex between VPS34ΔC2 and SB02024 reveals a structural organization similar to the one observed for the structure of apo‐VPS34 (PDB ID: 3IHY [21], r.m.s.d. = 0.55 Å for 537 aligned Cα). In the two VPS34 molecules present in the asymmetric unit, a region from the N‐terminal helical domain encompassing residues 418–471 could not be modeled due to the lack of electron density. The N‐terminal helical domain (light cyan ribbon, Fig. 1B) forms extensive contacts with both the N‐lobe (yellow ribbon, Fig. 1B) and the C‐lobe (pink ribbon, Fig. 1B) of the kinase domain burying 2360 and 1540 Å^2^ of solvent accessible surface upon interaction with the respective lobes of the kinase domain (Fig. 1B). Two α‐helices from the N‐terminal helical domain are sandwiched between the N‐ and C‐kinase domain lobes restricting the interlobular motion inherent to the protein kinase family. SB02024 resides in the ATP pocket active site of VPS34 between the two lobes of the kinase domain. Interaction between SB02024 and VPS34 is mainly governed by hydrogen bonds (either direct or water‐mediated), hydrophobic, and van der Waals forces (Fig. 1C). The oxygen atom on the morpholine ring of SB02024 forms a direct hydrogen bond with backbone nitrogen of hinge‐residue I685. The pyridone group of SB02024 is involved in two sets of water‐mediated hydrogen bonds. The pyridone oxygen carbonyl is bridged by two water molecules with residues K636, D644, and Y670 from the N‐lobe, while the pyridone nitrogen interacts through a water molecule with sidechain of D761 of the DFG motif present in the activation loop. Two exocyclic moieties, a trifluoromethyl and a methyl located, respectively, on the piperidine and the morpholine ring of SB02024 fill two hydrophobic cavities. The pocket occupied by the trifluoromethyl is delineated by three P‐loop residues (F612, S614, and P618) and three N‐lobes residues (I634, K636, and M682). The cavity occupied by the methyl group on the morpholine ring is formed by the sidechains of I634, M682, and hinge‐residue F684. The septum between these two hydrophobic pockets is created by the sidechains of I634 and M682. SB02024 occupancy of these two hydrophobic cavities and targeting side‐chains of M682 and F684, which are unique among phosphatidylinositol 3‐kinases, imparts greater potency and higher selectivity than its des‐trifluoromethyl or des‐methyl analogs, including greater selectivity versus other lipid kinase family members (Fig. S1) [16].

Next, the biochemical inhibition of VPS34 by SB02024 was measured using a time‐resolved fluorescence resonance energy transfer (FRET)‐based assay. SB02024 proved to be a highly potent VPS34 inhibitor with an IC_50_ of 5 nm (Fig. 1D). We further evaluated the inhibition of VPS34 activity in cells using a NanoBRET assay, affording a comparable IC_50_ of 5.7 nm (Fig. 1E). SB02024 profiling was then further extended to physiochemical and pharmacokinetic properties summarized in a table (Fig. 1F). SB02024 showed excellent solubility in both gastric and small intestinal pH media and exhibited moderate lipophilicity as measured by shake flask determination of Log*D* (1.8). SB02024 also exhibited excellent membrane permeability as determined in the CaCo2 monolayer assay without the liability of efflux (A → B 51 × 10^−6^ cm·s^−1^; Efflux Ratio 0.9). SB02024 exhibited low *in vitro* and *in vivo* clearance, weak inhibition of CYP3A, no induction of CYP3A4, and high plasma‐free fractions of 14% (mouse) and 44% (human). Finally, SB02024 demonstrated high oral bioavailability (96%) in a mouse PK study. Taken together, SB02024 constitutes a suitable candidate for *in vivo* studies.

### 3.2 SB02024 demonstrates target engagement *in vivo* in the H1299 tumor model

**Fig. 2:**
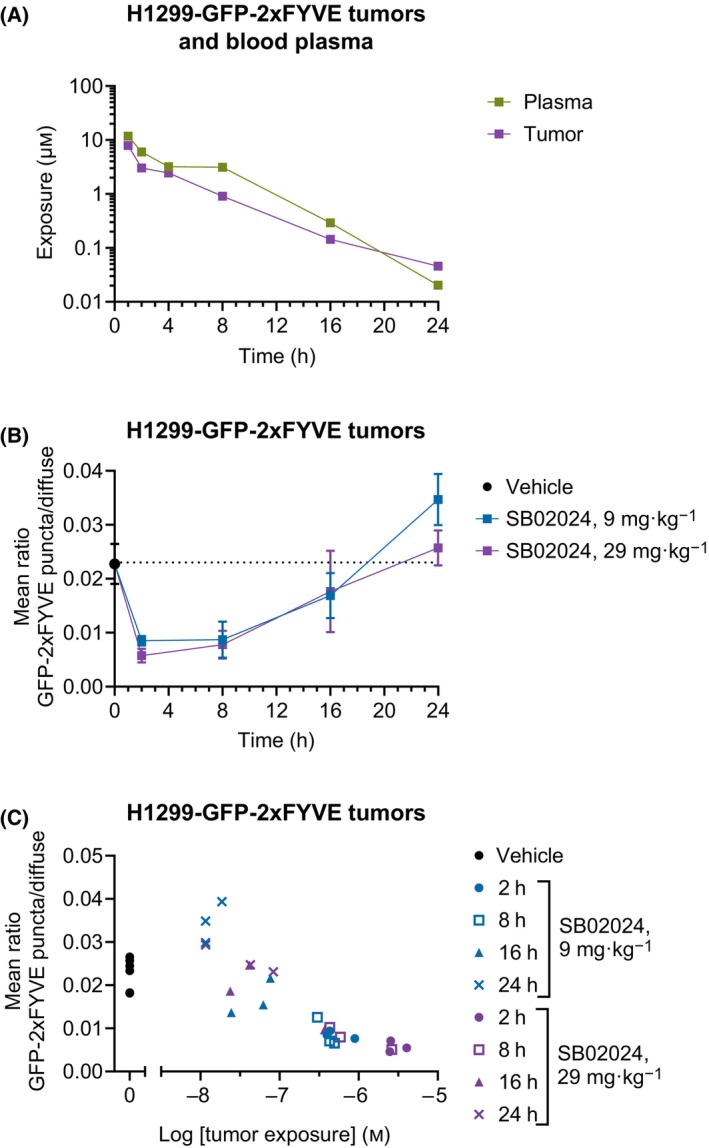
Pharmacokinetic and ‐dynamic profile of SB02024. (A–C) H1299 cells expressing GFP‐2xFVYE were subcutaneously implanted in NOD.Cg‐Prkdc^*scid*^ Il2rg^*tm1Sug*^/JicTac mice. Mice were administered with one dose of vehicle (*n* = 6), 9 mg·kg^−1^ (*n* = 18) or 29 mg·kg^−1^ (*n* = 18) of SB02024 by oral gavage. (A) Concentration of SB02024 in blood plasma (green) and tumor tissue (purple) of *n* = 3 mice at indicated time points after one dose of 29 mg·kg^−1^. (B) Quantification VPS34 activity in tumors at indicated timepoints and dose as measured by anti‐GFP staining of GFP‐2xFYVE puncta using immunohistochemistry. Data represent mean ratio ± SD of *n* = 3 mice per time point for SB02024 treatment and *n* = 6 mice for vehicle treatment (plotted for reference at *t* = 0 h). The dotted line indicates signal mean for vehicle control animal. (C) Correlation of GFP‐2xFYVE puncta and SB02024 concentration in each individual tumor at indicated time point and dose as compared to vehicle control tumors.

Next, we performed pharmacokinetic and pharmacodynamic (PK/PD) profiling of SB02024 in tumor‐bearing mice. CIEA NOG mice were subcutaneously implanted with H1299 cells expressing GFP‐2xFVYE. The FVYE domain binds specifically to phosphatidylinositol‐3‐phosphate (PI3P), the product of VPS34 kinase activity, and thus allows for the determination of VPS34‐produced PI3P intracellular localization at autophagosome/endosome puncta *in vitro* and *in vivo* [22]. We previously determined the efficacy of SB02024 on VPS34 inhibition in these cells *in vitro* [16]. Oral administration of 29 mg·kg^−1^ SB02024 resulted in a rapid increase of both plasma and tumor exposure, with a *C*_max_ of around 10 μm after 2 h. Both tumor and plasma concentrations decreased linearly with time with *t*_1/2_ = 2.5 h (Fig. 2A). Treatment with 9 or 29 mg·kg^−1^ SB02024 (doses empirically determined) inhibited GFP‐2xFYVE puncta (autophagosome and endosome) formation in H1299 tumors after 2 h and the effect lasted until about 8 h (Fig. 2B) which correlated well with the tumor concentration of SB02024 (Fig. 2C). Together, these data show tumor target engagement of SB02024 and highlights its suitability for evaluating VPS34 inhibition in tumor xenograft studies.

### 3.3 Inhibition of VPS34 activates an anti‐tumor immune response via IFN signaling in a renal cancer model

**Fig. 3:**
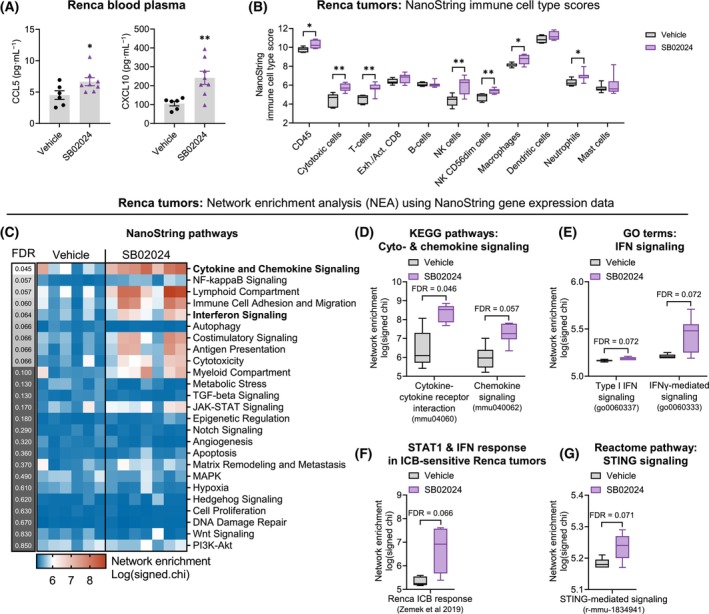
VPS34i treatment increases an anti‐tumor immune response via IFN signaling *in vivo*. (A) Quantification of CCL5 and CXCL10 protein levels in the blood plasma of Renca tumor‐bearing BALB/c mice treated with control vehicle (*n* = 6) or 20 mg·kg^−1^ SB02024 (*n* = 8) using Mesoscale Discovery assays. (B) NanoString gene signature analysis for immune cell types in Renca tumors treated with either control vehicle (vehicle, *n* = 6) or 20 mg·kg^−1^ SB02024 (*n* = 7). NK, natural killer. (A, B) Graphs represent mean ± SEM. *P < 0.05; **P < 0.01 using an unpaired two‐tailed Student's *t*‐test. (C–G) NEA was carried out using NanoString gene expression data. One‐way ANOVA test was performed with FDR < 0.1 as a cut‐off threshold for statistical significance. (C) Heatmap showing log(signed chi) values for enrichment of NanoString gene signatures for immune pathways. Blue, low enrichment; red, high enrichment. JAK, janus kinase; MAPK, mitogen‐activated protein kinase; NF, nuclear factor; PI3K, phosphoinositide 3‐kinase; TGF, transforming growth factor. (D–G) Boxplots showing log(signed chi) values for enrichment of (D) KEGG pathways involved in cytokine signaling (mmu04060, mmu04062), (E) GO terms related to interferon signaling (go0060337, go0060333), (F) a gene signature for ICB sensitivity in Renca published by Zemek et al. [25], and (G) reactome pathway for STING signaling (r‐mmu‐1834941).

We previously demonstrated that VPS34 inhibition reduces tumor growth in murine cancer models of melanoma, CRC, and renal cell carcinoma (RCC) [18]. In melanoma and CRC tumor models, the effects are NK and CD8^+^ T cell‐dependent and associated with increased levels of chemokines CCL5 and CXCL10. Here we further investigated if a similar anti‐tumor immune response is activated in RCC by treating Renca tumor‐bearing mice with the VPS34i SB02024. Consistent with our previous data, we detected a significant increase in CCL5 and CXCL10 in the blood plasma of mice treated with 20 mg·kg^−1^ SB02024, indicating the activation of a proinflammatory immune response (Fig. 3A).

To better understand the impact of VPS34i on the tumor immune landscape and identify key mechanisms involved in the chemokine induction, we assessed mRNA expression levels from Renca tumors, with a pre‐selected list of 750 genes involved in the tumor development, microenvironment, or immune response (NanoString immune‐oncology panel). This profiling revealed significantly increased gene expression of protein tyrosine phosphatase receptor type c (*Ptprc*) associated with CD45^+^ cells upon SB02024 treatment (Fig. 3B). Further profiling of immune cell subsets showed a significant increase in gene signatures characteristic for cytotoxic cells, T cells, both total and CD56^dim^ NK cells, macrophages, and neutrophils (Fig. 3B). Together, these results indicate that VPS34i treatment modulates the tumor immune landscape in renal cancer, confirming our previous findings in melanoma and CRC [18].

To gain more mechanistic insight into the impact of VPS34i treatment on Renca tumors, we used network enrichment analysis (NEA) [23] which confirmed that, upon treatment, cytokine and chemokine signaling was the most strongly altered among the 25 signature gene sets annotated by NanoString (Fig. 3C). Similarly, among pathways of the KEGG database, we found cytokine and chemokine signaling (mmu04060, mmu040062) as significantly differentially activated (Fig. 3D). Among the NanoString gene sets, IFN signaling was another pathway highly enriched upon VPS34 inhibition (Fig. 3C). This was further confirmed by a significant enrichment of respective gene ontology (GO) terms for mouse type I and type II IFN signaling (go0060337, go0060333) (Fig. 3E). Canonical IFN signaling is known to activate signal transducer and activator of transcription (STAT) pathway, resulting in transcription of IFN‐stimulated genes (ISGs) [24]. We previously discovered that interferon regulatory factor 7 (IRF7) and STAT1 are involved in the proinflammatory cytokine response induced by SB02024 and reported increased IFNγ levels in the TME [18]. A previous study reported a STAT1 and IFN gene signature crucial for sensitivity to immune checkpoint blockade (ICB) in the Renca model [25]. We found that this gene signature was significantly enriched in SB02024‐treated tumors as compared to vehicle controls (Fig. 3F). Type I IFN signaling can in turn be induced via the cytosolic DNA‐sensing cyclic GMP‐AMP synthase (cGAS)‐stimulator of interferon genes protein (STING) pathway. Recent studies suggest crosstalk between autophagy, endosomal trafficking, and the cGAS‐STING pathway [26, 27]. Indeed, we discovered enrichment of the Reactome gene signature for STING‐mediated signaling (r‐mmu‐3134975) in the SB02024‐treated tumors as compared to vehicle controls indicating involvement of the cGAS‐STING pathway (Fig. 3G). In summary, this analysis demonstrated that pharmacological VPS34 inhibition in renal cancer leads to an anti‐tumor immune response characterized by proinflammatory cytokine/chemokine release and increased expression of IFN response genes.

### 3.4 Type I IFN response and increased chemokine secretion with VPS34 inhibitors is cGAS/STING‐dependent

**Fig. 4:**
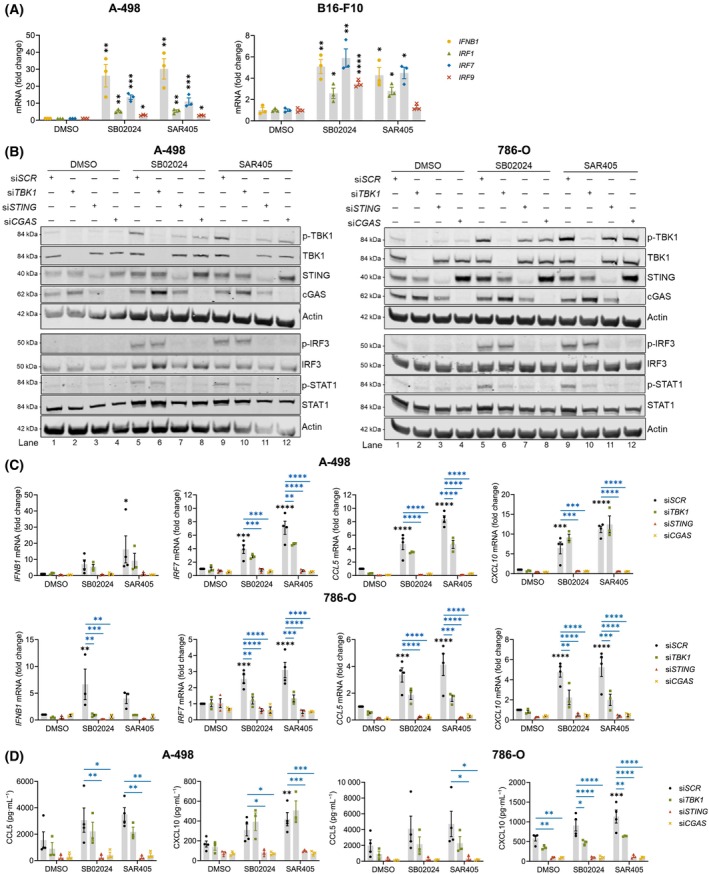
VPS34 inhibition triggers type I IFN signaling in a cGAS/STING‐dependent manner *in vitro*. (A) Quantification of *IFNB1*, *IRF1*, *IRF7*, and *IRF9* gene expression after treatment with DMSO control or 2 μm of VPS34 inhibitor (= VPS34i, SB02024 or SAR405) for 24 h in A‐498 or B16‐F10 cells using qRT‐PCR. Bars represent mean ± SEM of three independent experiments. (B–D) A498 or 786‐O cells were transfected with scrambled siRNA control (siSCR) or siRNA targeting *TBK1* (si*TBK1*), *STING* (si*STING*) or *CGAS* (si*CGAS*) for 48 h and then treated as in panel A. (B) Western blot images showing expression of indicated proteins. Upper and lower blot for each cell line were derived from the same lysate and performed simultaneously. Blots are representative of three independent experiments. (C) Quantification of *IFNB1*, *IRF7*, *CCL5*, or *CXCL10* gene expression using qRT‐PCR. Bars represent mean ± SEM of four (si*SCR*) or *three* (si*TBK1*, si*STING*, si*CGAS*) independent experiments. (D) Quantification of CCL5 or CXCL10 secretion in media supernatant using mesoscale discovery assays. Bars represent mean ± SEM of four (si*SCR*) or three (si*TBK1*, si*STING*, si*CGAS*) independent experiments. *P < 0.05; **P < 0.01; ***P < 0.001; ****P < 0.0001 using one‐way ANOVA followed by Dunnett's (A) or Šidák multiple comparison test (C, D; black asterisks indicate comparison to DMSO‐treated si*SCR* control and blue to indicated VPS34i‐treated si*SCR* control).

To further investigate whether VPS34 inhibition activates a type I IFN response in cancer cells, we treated human RCC A‐498 and murine melanoma B16‐F10 cells with VPS34i SB02024 or SAR405 [12]. We observed significantly increased transcription of *IFNB1* and the Interferon Stimulated Genes (ISGs) IRF1, *IRF7*, and *IRF9* (Fig. 4A). Treatment with SB02024 or SAR405 also significantly increased *IFNB1* expression in human melanoma Me30966 cells (Fig. S2A).

We next investigated whether this may occur due to activation of the cGAS‐STING pathway as indicated by the NEA of SB02024‐treated tumors (Fig. 3G). Upon activation of STING, tank‐binding kinase 1 (TBK1) is phosphorylated which in turn phosphorylates transcription factor IRF3 [28]. VPS34i treatment of A‐498 or 786‐O cells induced TBK1 and IRF3 phosphorylation (lanes 5 and 9), indicating activation of the STING pathway (Fig. 4B). To assess the functional role of the cGAS‐STING pathway, we silenced TBK1, STING, or cGAS expression using siRNA transfection (si*TBK1*, si*STING*, and si*CGAS*, respectively). While si*TBK1* did not affect IRF3 and STAT1 phosphorylation (lanes 6 and 10), either si*STING* or si*CGAS* completely abrogated VPS34i‐induced IRF3 and STAT1 phosphorylation (lanes 7, 8, 11, and 12) (Fig. 4B). These results clearly show that the cGAS‐STING pathway is activated upon VPS34i treatment and is responsible for the activation of IFN signaling. Of note, si*STING* or si*CGAS* only had a limited impact on phospho‐TBK1 levels (lane 7, 8, 11, and 12), suggesting that other innate immune signaling pathways might also be triggered upon VPS34i treatment. VPS34i treatment also induced expression of IRF3 target genes (*IFNB1*, *IRF7*, *CCL5*, and *CXCL10*) [29, 30] in A‐498 and 786‐O cells incubated with si*SCR* control (Fig. 4C). Interestingly, the VPS34i‐dependent increase in IRF3 target gene expression was only slightly decreased by si*TBK1* but significantly decreased by si*STING* or si*CGAS* (Fig. 4C), indicating a cGAS/STING‐dependency. CCL5 and CXCL10 secretion was induced by the VPS34i in A‐498 and 786‐O cells, and RNAi targeting cGAS or STING inhibited this secretion significantly (Fig. 4D).

The activation of the STING pathway by VPS34 inhibition was not limited to the RCC cells but similarly observed in the Me30966 melanoma cell line as evidenced by increased IRF3 and STAT1 phosphorylation (lanes 3 and 5) in a STING‐dependent manner (lanes 4 and 6) (Fig. S2B). Furthermore, we found that VPS34i‐induced *IFNB1*, *CCL5*, and *CXCL10* expression, with some variation in the data, is also STING‐dependent in Me30966 cells (Fig. S2C).

To confirm that the activation of the cGAS‐STING pathway depends on VPS34 and is not an off‐target of VPS34i, we silenced the VPS34 gene by siRNA and found a significant increase in IRF3 phosphorylation in A‐498 cells (Fig. S3A,B, lane 4). STAT1 phosphorylation also increased approximately fourfold but was not statistically significant (Fig. S3A,B, lane 4). Notably, si*VPS34* treatment resulted in a more pronounced increase of STING levels (Fig. S3A,B, lane 4) as compared to VPS34i treatment (Fig. 4B, lanes 5 and 9). This suggests that under normal conditions VPS34 may control cellular STING levels, and inhibition of VPS34 may cause STING accumulation and activation. Furthermore, si*VPS34* significantly induced expression of IRF3 target genes *IFNB1*, *IRF7*, and *CCL5* and showed a clear trend to increase *CXCL10* (Fig. S3C). This also translated into a significantly increased secretion of IFNβ, CCL5, and CXCL10 proteins (Fig. S3D) indicating activation of a type I IFN response. To prove that also si*VPS34*‐induced type I IFN response activation is cGAS/STING‐dependent, we performed a tandem knockdown by silencing either STING or cGAS by siRNA before si*VPS34* knockdown. Phospho‐IRF3 and STING levels induced by si*VPS34* were significantly decreased with cGAS knockdown (Fig. S3A, lane 6). si*STING* had a more heterogeneous effect (Fig. S3A, lane 5), which could be explained with the better knockdown efficiency of si*CGAS* in these experiments (Fig. S3A,B, compare STING and cGAS quantification). However, siVPS34‐induced IFNβ expression and secretion were dependent on both si*STING* and si*CGAS* (Fig. S3C,D). Altogether, we uncover that both pharmacological and siRNA‐mediated VPS34 inhibition in RCC and melanoma cells activates the type I IFN and proinflammatory cytokine response via induction of the cGAS‐STING pathway.

### 3.5 VPS34 inhibition in combination with STING agonism enhances proinflammatory cytokine responses in cancer and innate immune cells

**Fig. 5:**
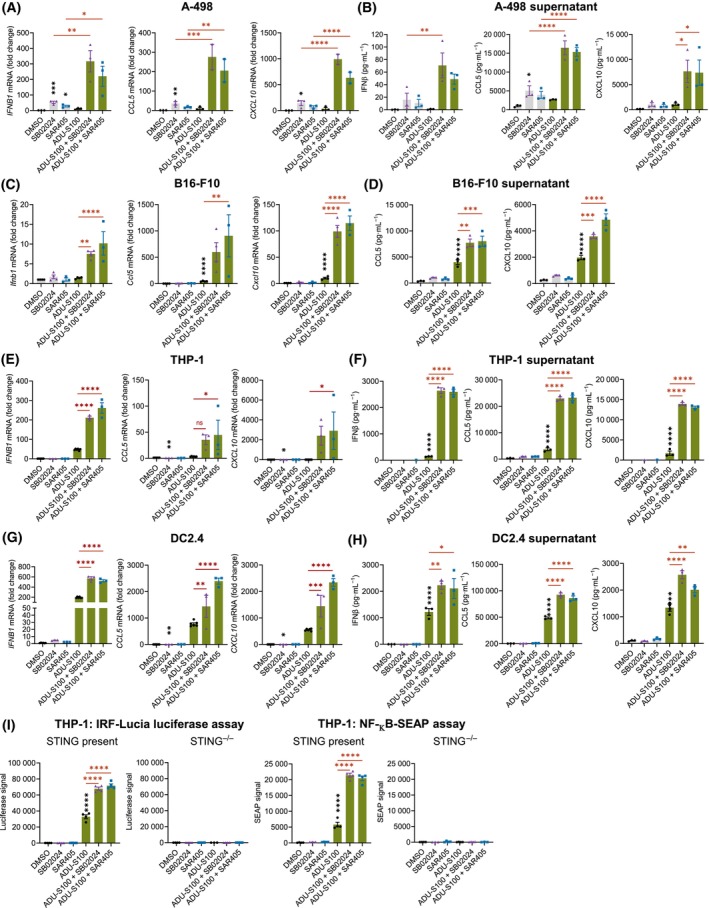
VPS34 inhibitors synergize with the STING agonist ADU‐S100 *in vitro*. (A) Quantification of *IFNB1*, *CCL5*, and *CXCL10* gene expression using qRT‐PCR and (B) secreted IFNβ, CCL5, and CXCL10 using Mesoscale Discovery (MSD) assays in A‐498 cells treated with DMSO control, 2 μm SB02024 or SAR405 in combination with 50 μm STING agonist ADU‐S100 for 24 h. Bars represent mean ± SEM of three independent experiments. (C) Quantification of *Ifnb1*, *Ccl5*, and *Cxcl10* gene expression using qRT‐PCR and (D) secreted CCL5 and CXCL10 using MSD assays in B16‐F10 cells treated with DMSO control, 2 μm SB02024 or SAR405 in combination with 5 μm ADU‐S100 for 24 h. Bars represent mean ± SEM of three independent experiments (in (C) four independent experiments were run for SB02024/ADI100 combo). (E) Quantification of *IFNB1*, *CCL5*, and *CXCL10* gene expression using qRT‐PCR and (F) secreted IFNβ, CCL5, and CXCL10 using ELISA assays in THP‐1 cells treated with DMSO control, 2 μm SB02024 or SAR405 in combination with 50 μm ADU‐S100 for 24 h. Bars represent mean ± SEM of three independent experiments. (G) Quantification of *Ifnb1*, *Ccl5*, and *Cxcl10* gene expression using qRT‐PCR and (H) secreted IFNβ, CCL5, and CXCL10 using ELISA assays in DC2.4 cells treated with DMSO control, 2 μm SB02024 or SAR405 in combination with 10 μm ADU‐S100 for 24 h. Bars represent mean ± SEM of three independent experiments. (I) THP‐1 reporter cells (STING present or knockout) were treated with DMSO, 2 μm of VPS34 inhibitor (SB02024 or SAR405) in combination with 5 μg·mL^−1^ ADU‐S100 for 24 h. Interferon‐stimulated response element (ISRE) or NFκB signal was read out via luciferase or secreted embryonic alkaline phosphatase (SEAP), respectively. Bars represent mean ± SEM of four independent experiments. *P < 0.05; **P < 0.01; ***P < 0.001; ****P < 0.0001 using one‐way ANOVA followed by Dunnett's (black asterisks indicating comparison to DMSO‐treated control) or Šidák multiple comparison test (red asterisks indicating comparison of the combination treatment to the more effective single treatment).

Activating the STING‐dependent cytokine response has received considerable attention in efforts to increase the anti‐tumor efficacy of immunotherapy. Several STING agonists have since entered clinical studies in cancer patients in combination with ICB. ADU‐S100, a synthetic analog of 2′,3′‐cyclic GMP‐AMP (cGAMP), was the first to enter clinical trials but demonstrated only limited anti‐tumor efficacy [31]. Here, we used ADU‐S100 as a tool compound to evaluate whether VPS34i treatment could improve proinflammatory cytokine responses toward STING agonists in cancer cells *in vitro*. Combination treatment of VPS34i (SB02024 or SAR405) with ADU‐S100 in A‐498 cells showed a significant increase of *IFNB1*, *CCL5*, and *CXCL10* gene expression and protein secretion as compared to single treatments (Fig. 5A,B). VPS34i/ADU‐S100 combination treatment in B16‐F10 cells also resulted in a significant increase of *Ifnb1* expression which was associated with a significant increase in *Ccl5* and *Cxcl10* expression and secretion as compared to single treatment (Fig. 5C,D). Similarly, enhanced proinflammatory cytokine responses following VPS34/ADU‐S100 combination treatment were observed in the human THP‐1 monocytic cell line and the murine DC2.4 dendritic cell line demonstrating these effects extend to both human and murine antigen‐presenting cells (Fig. 5E–H). To further investigate if the observed effects are STING‐dependent, we tested the VPS34i/ADU‐S100 combination treatment in THP1‐Dual™ cells (IRF‐Lucia luciferase and NF‐κB‐SEAP reporter) in the presence or absence of STING. VPS34i/ADU‐S100 combination treatment significantly increased activation of both reporters as compared to ADU‐S100 only in THP1‐Dual™ cells with STING present (Fig. 5I), indicating STING‐dependent activation of the IRF and NF‐κB pathway. As a further confirmation, combination treatment with the natural STING agonist cGAMP, an alternative way to activate STING, resulted in a similar increase of *Ccl5* and *Cxcl10* expression (Fig. S4A) as well as CXCL10 secretion while having no significant effect on CCL5 secretion in CT26, 4T1, YUMM1.7 cells (Fig. S4B).

Next, we tested whether the proinflammatory cytokine response to the combination treatment requires functional autophagy by knocking down *autophagy related 5* (*Atg5*), a crucial autophagy gene. *Atg5* siRNA efficiently suppressed *Atg5* expression (Fig. S5A), significantly increased *Ccl5* expression, and demonstrated a tendency to increase *Cxcl10* expression in B16‐F10 cells (Fig. S5B). VPS34i treatment maintained its effectiveness in inducing the expression of *Ccl5* in B16‐F10 cells, even when transfected with *Atg5* siRNA. Importantly, the STING agonist ADU‐S100 continued to synergize with VPS34i to enhance *Ccl5* expression in cells transfected with *Atg5* siRNA (Fig. S5C). These findings suggest that the capacity of VPS34 inhibitors, whether applied alone or in combination with the STING agonist, to induce a proinflammatory cytokine response is only partially independent on autophagy.

We then conducted additional experiments to understand the dynamics of the STING pathway activation when using the combination treatment of VPS34 and ADU‐S100. Incubation of B16‐F10 or DC 2.4 cells with the VPS34/ADU‐S100 combination treatment resulted in longer activation of the STING pathway compared to single agent treatment as evidenced by prolonged STING (upper band) and TBK1 phosphorylation (Fig. S6A–C). This finding implies that the extended activation of the STING pathway may be the underlying mechanism responsible for the heightened downstream IFN signaling observed when combining VPS34 inhibition with STING agonism. Taken together, these data collectively suggest that VPS34 inhibition can synergize with STING agonists in promoting a proinflammatory cytokine response in both cancer and innate immune cells in both an autophagy‐dependent and ‐independent manner.

### 3.6 SB02024 treatment improves the efficacy of STING agonist ADU‐S100 *in vivo*

**Fig. 6:**
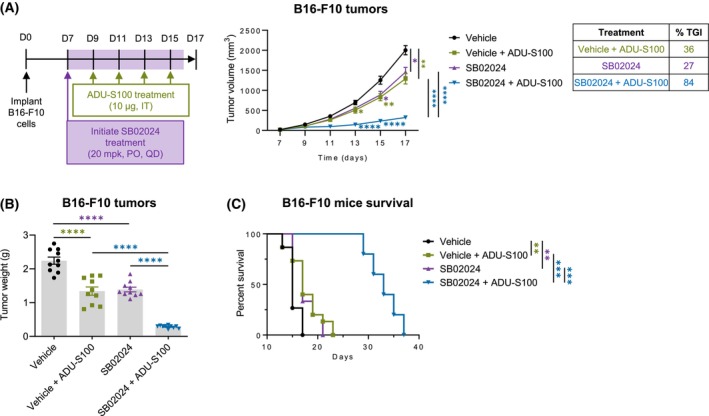
SB02024 potentiates the response to STING agonist in tumor‐bearing mice. (A–C) B16‐F10 cells were subcutaneously implanted in C57BL/6 mice. Mice were injected intratumorally with PBS or 10 μg STING agonist ADU‐S100 (on days 9, 11, 13, and 15) in combination with daily oral administration of vehicle or 20 mg·kg^−1^ SB02024 by gavage starting from day 7 to day 16 after implantation. (A) Dosing schematic and tumor growth curves of *n* = 15 mice per treatment group. Percent tumor growth inhibition was calculated compared to vehicle control. (B) Tumor weight of *n* = 10 mice per treatment group sacrificed on day 17. (C) Mice survival of *n* = 5 mice per treatment group. Lack of survival was defined as death or tumor size > 1000 mm^3^. Data represent mean ± SEM. *P < 0.05; **P < 0.01; ***P < 0.001; ****P < 0.0001 using one‐way ANOVA followed by Šidák multiple comparison test. Survival percentage was defined using graphpad prism, and *P* values were calculated using the log‐rank (Mantel‐Cox) test.

Following our *in vitro* findings that B16‐F10 cells synergistically increased proinflammatory cytokine release, we sought to assess the therapeutic benefit of combining VPS34i SB02024 with STING agonist ADU‐S100 in B16‐F10 tumor‐bearing mice. SB02024 was dosed as previously described [18] and ADU‐S100 was injected intratumorally at a suboptimal dose (4 × 10 μg) based on published data [32]. Combination treatment showed significantly decreased tumor growth and tumor weight (Fig. 6A,B) along with a significantly improved survival benefit as compared to each of the single treatments in B16‐F10 tumor‐bearing mice (Fig. 6C). These data demonstrate that VPS34i can render tumors more responsive to STING agonist treatment and provide significant increases in survival. To assess the clinical significance of activating the STING pathway in melanoma patients, we utilized a publicly available dataset of melanoma patients. A total of 448 skin cutaneous melanoma samples from the TCGA PanCancer Atlas were categorized into two groups based on their expression levels of *cGAS*: high and low. Our findings (Fig. S7A) reveal a significant survival advantage among melanoma patients with high *cGAS* expression compared to those with lower *cGAS* levels. This underscores the therapeutic potential of enhancing the STING pathway using STING agonists in the context of melanoma. Expanding upon these clinical observations, we have demonstrated that melanoma patients with elevated *cGAS* levels also exhibit increased expression of *CCL5* and *CXCL10*, as illustrated in (Fig. S7B, upper panels). These results are consistent with our *in vitro* findings using the B16‐F10 melanoma model. Additionally, we analyzed the immune infiltration patterns in melanoma patients with high and low *cGAS* expression levels. Our data (Fig. S7C, middle and lower panels) revealed heightened expression of CD8^+^ T cell markers (*CD8a* and *CD8b*) and NK cell markers (*NCR1* and *NCR3*) in melanoma patients with elevated levels of *cGAS*/*CCL5*/*CXCL10*. This provides valuable insights into the role of modulating the STING pathway in recruiting cytotoxic immune cells. Collectively, these findings support the concept that enhancing the STING pathway leads to increased infiltration of CD8^+^ T and NK cells while reducing its activity produces the opposite effect.

## 4 Discussion

While novel immunotherapies have developed into a powerful addition to the anti‐cancer therapy toolbox, a majority of patients remain unresponsive [33]. This has ignited the field to identify underlying immune evasion mechanisms to improve clinical responses. An intact type I IFN response underlies the efficacy of various anti‐cancer therapies including radiotherapy, chemotherapy, and immunotherapies [34]. Herein, we demonstrate that inhibiting VPS34, a lipid kinase essential for vesicle trafficking and autophagy, activates type I and type II IFN response gene expression, increases CCL5 and CXCL10 release in blood plasma, and promotes effector immune cell infiltration in Renca tumor‐bearing mice. In addition, VPS34 inhibition also increases the IFN response in several cancer and myeloid cell lines in combination with a STING agonist. Our results are supported by a recent study showing that conditional whole‐body deletion of the autophagy gene *Atg7* induces a type I and II IFN response in urothelial tumors resulting in the release of inflammatory cytokines and anti‐tumor immunity induction. The study further demonstrated that tumor growth inhibition in *Atg7*‐deficient hosts is STING‐dependent [5]. This is consistent with our findings that the VPS34i‐induced type I IFN response is dependent on the cGAS‐STING pathway in both RCC and melanoma cancer cells and myeloid cells. However, the precise mechanism tying VPS34 inhibition to the cGAS/STING pathway remains to be elucidated. The STING protein is degraded via autophagy and/or endosomal trafficking to lysosomes [26, 27, 35], processes which are both affected by VPS34 inhibition. Indeed, we observed significant STING accumulation following VPS34 knockdown and slight STING accumulation following VPS34i treatment in cancer cells. Our data support a model where VPS34‐mediated inhibition of both autophagy and endosomal trafficking contribute to the activation of the cGAS/STING pathway. The activation is still observed in the background of autophagy knockdown, and combining STING agonist with VPS34i prolonged the activation of the STING pathway (measured as the presence of STING and TBK1 phosphorylated forms). Furthermore, based on our observation that VPS34i‐dependent induction of IFN response relies on cGAS, it is tempting to speculate that cytosolic DNA accumulation could be a major upstream event in these settings. This could imply that another component of the MOA of VPS34i may inhibit autophagic/endosomal clearance of cytosolic DNA. Mitochondrial DNA accumulation was shown to activate the STING pathway when combining autophagy inhibition (by hydroxychloroquine treatment or knockout of *Atg5* or *Atg7*) with irradiation in breast cancer models [36]. VPS34i‐induced cGAS‐STING signaling may also be the underlying MOA of the sensitization to anti‐PD‐1/‐PD‐L1 therapy in melanoma and CRC models as previously reported by us [18]. Therefore, combining treatment with a VPS34i may improve the therapeutic benefit of an array of immunotherapeutic agents.

Pharmacological activation of the STING pathway using STING agonists has been introduced as an approach to improve the outcome of ICB. Intratumoral ADU‐S100 injections elicit a local IFN response leading to robust anti‐tumor immune responses in preclinical models but demonstrated only limited clinical efficacy highlighting the need to improve these combinatorial approaches [31]. In addition, STING agonists like ADU‐S100 are limited in their use to injectable tumors and may not address metastatic disease. Thus, the development of systemic or tumor‐targeted STING agonists is warranted and already underway [37]. Orally administered pharmacological VPS34i are well tolerated in mice and can increase immunogenicity in a T and NK cell‐dependent manner, as we have previously shown [18]. While the effects of the ADU‐S100/VPS34i combination on immune effector cells remain to be investigated, we reported a significant increase in proinflammatory cytokine response in various cancer cell lines upon combination of VPS34i with STING agonists. These effects may enhance immune cell recruitment and activation in the TME.

We further demonstrate that the ADU‐S100/VPS34i combination significantly inhibited tumor growth and increased mice survival in B16‐F10 tumor‐bearing mice. These data indicate that systemic pharmacological inhibition of autophagy and/or endosomal trafficking increases sensitivity to STING‐targeting therapies. Supporting this concept, Gonugunta et al. [27] showed that combination treatment of cGAMP with Bafilomycin A1 significantly increased C10 expression and decreases tumor growth in the B16 melanoma model. Likewise, emerging evidence supports targeting autophagy to modulate responses to cancer immunotherapies. Autophagy deficiency was shown to sensitize cancer cells to T cell killing [9] and increases response to immunotherapy *in vivo* [38]. Autophagy inhibition using small molecule inhibitors of ULK1 or PIKfyve was shown to increase responses to ICB in tumor‐bearing mice [39, 40].

## 5 Conclusions

In summary, our study reveals that VPS34 inhibition activates an inflammatory IFN response via activation of the cGAS‐STING pathway. We have demonstrated that combining VPS34i with STING agonist treatment enhances a STING‐dependent IFN response *in vitro*. The increased anti‐tumor efficacy of ADU‐S100 in combination treatment with VPS34i *in vivo* further suggests that pharmacological VPS34 inhibition may be a promising strategy to increase immune cell infiltration and improve responses to existing and emerging cancer immunotherapies.

## Conflict of interest

YY, SP, LT, CS, JL, JV, MA, JM, and ADM are employees/shareholders of Sprint Bioscience. MB, JS, MJT, HA, BDS, and DLF are employees/shareholders of Deciphera Pharmaceuticals. The other authors declare no potential conflicts of interest.

## Author contributions

YY, MB, MZM, EB, KVM, SP, LT, CS, JL, BDS, DLF, JV, MA, JM, KPT, ADM, and BJ contributed to study concept and experimental design; YY, MB, MZM, SP, EB, KVM, SCK, KBS, LT, CS, JL, JS, MJT, HA, BDS, and JV contributed to data curation; YY, MB, MZM, SP, LT, CS, JL, BDS, AA, and JV contributed to data analysis and interpretation; YY, MB, BDS, DLF, MA, JM, KPT, ADM, and BJ contributed to study supervision and administration; DLF, MA, JM, KPT, ADM, and BJ contributed to resources and funding acquisition; YY, MB, and LT contributed to writing the manuscript; YY, MB, MZM, SP, LT, BDS, DLF, MA, KPT, ADM, and BJ contributed to editing the manuscript.

### Peer review

The peer review history for this article is available at https://www.webofscience.com/api/gateway/wos/peer‐review/10.1002/1878‐0261.13619.

## Supporting information


**Fig. S1.** Sequence alignment of kinase domains of human phosphatidylinositol 3‐kinases.
**Fig. S2.** VPS34 inhibition triggers STING‐dependent proinflammatory response in human melanoma cells Me30966.
**Fig. S3.** Knockdown of VPS34 phenocopies increased type I IFN signaling and its cGAS/STING‐dependency.
**Fig. S4.** VPS34i synergize with STING ligand cGAMP *in vitro*.
**Fig. S5.** Proinflammatory cytokine response induced by VPS34 inhibitor/ADU‐S100 combination treatment is also observed in the background of *Atg5* knockdown.
**Fig. S6.** VPS34 inhibitor SB02024 combined with STING agonist ADU‐S100 induces prolonged activation of cGAS‐STING pathway.
**Fig. S7.** Assessing the value of activating STING pathway in melanoma patients.

**Table S1.** Data collection and refinement statistics for crystal structure.
**Table S2.** Network enrichment analysis (NEA) network generation.
**Table S3.** List of used siRNAs, antibodies, and RT‐qPCR primer sequences.
**Table S4.** Overall survival (OS) information of the melanoma patients expressing high and low *cGAS*.
**Table S5.** Disease‐specific survival (DSS) information of the melanoma patients expressing high and low *cGAS*.
**Table S6.** Expression values of *CCL5*, *CXCL10*, *CD8A*, *CD8B*, *NCR1*, and *NCR3* of the melanoma patients expressing high and low *cGAS*.

## Data Availability

All data needed to evaluate the conclusions in the paper are present in the paper and/or the [Link], [Link]. Additional data related to this paper may be requested from the authors. Structure of VPS34ΔC2/SB02024 complex has been deposited under the PDB accession code 8RXR.
