# Supplementary material for: Combining VPS34 inhibitors with STING agonists enhances type I interferon signaling and anti‐tumor efficacy
Source: Mol Oncol. 2024 Mar 20;18(8):1904–22. doi: 10.1002/1878-0261.13619 (PMC11306511; doi:10.1002/1878-0261.13619)
Supplement: Supplementary file 1 — Fig. S1. Sequence alignment of kinase domains of human phosphatidylinositol 3‐kinases. Fig. S2. VPS34 inhibition triggers STING‐dependent proinflammatory response in human melanoma cells Me30966. Fig. S3. Knockdown of VPS34 phenocopies increased type I IFN signaling and its cGAS/STING‐dependency. Fig. S4. VPS34i synergize with STING ligand cGAMP in vitro. Fig. S5. Proinflammatory cytokine response induced by VPS34 inhibitor/ADU‐S100 combination treatment is also observed in the background of Atg5 knockdown. Fig. S6. VPS34 inhibitor SB02024 combined with STING agonist ADU‐S100 induces prolonged activation of cGAS‐STING pathway. Fig. S7. Assessing the value of activating STING pathway in melanoma patients. [file MOL2-18-1904-s002.pdf]

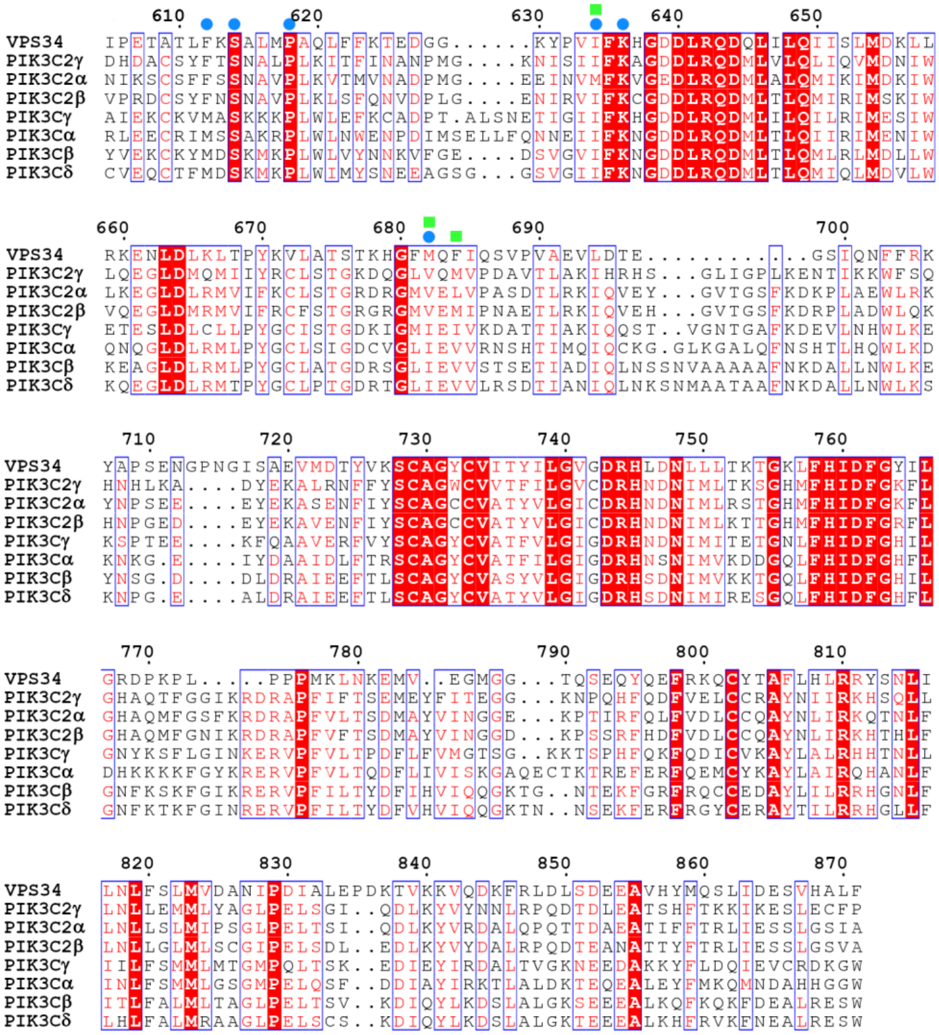

Supplementary Figure 1. Sequence alignment of kinase domains of human phosphatidylinositol 3-kinases

The numbering corresponds to VPS34 sequence. Residues lining the hydrophobic cavities accommodating the trifluoromethyl and the methyl groups of SB02024 are indicated by a blue disc and a green square, respectively. Alignment was performed using Clustal Omega and colored according to the conservation of the biophysical properties across sequences by ESPript 3.0 [1,2].

References:

1 Madeira F, Pearce M, Tivey ARN, et al. Search and sequence analysis tools services from EMBL-EBI in 2022. Nucleic Acids Res 2022;:gkac240. doi:10.1093/nar/gkac240

2 Robert X, Gouet P. Deciphering key features in protein structures with the new ENDscript server. Nucleic Acids Res 2014;42:W320–4. doi:10.1093/nar/gku316

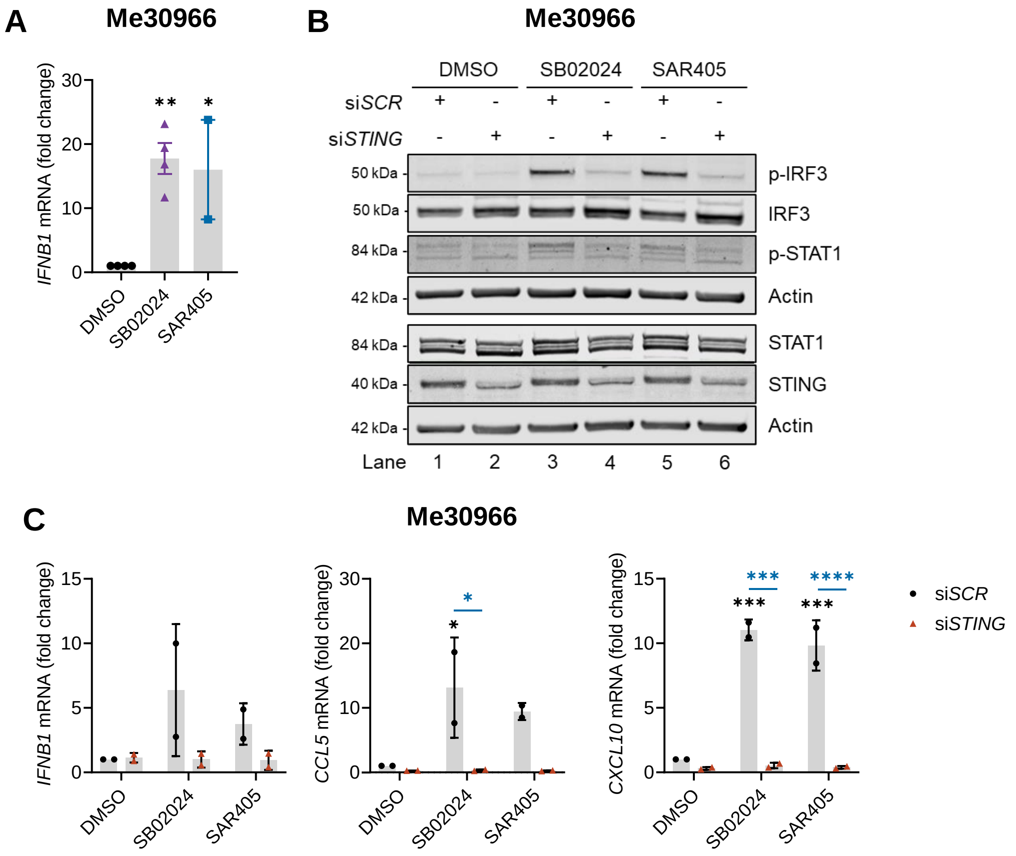

Supplementary Figure 2. VPS34 inhibition triggers STING-dependent proinflammatory response in human melanoma cells Me30966

(A) *IFNB1* gene expression quantified by qRT-PCR after 24h treatment with DMSO control or 2 µM of VPS34 inhibitor (=VPS34i, SB02024 or SAR405) in Me30966 cells. **(B, C)** Me30966 cells transfected with scrambled siRNA control (si*SCR*) or siRNA targeting STING (si*STING*) for 48h, then treated as in panel A. **(B)** Western blot of indicated proteins. Upper and lower blot were derived from the same lysate and performed simultaneously. Blot is representative of two independent experiments. **(C)** Quantification of *IFNB1*, *CCL5* or *CXCL10* gene expression using qRT-PCR. Data represent mean ± SEM of the indicated sample numbers. * $p < 0.05$; ** $p < 0.01$; *** $p < 0.001$; **** $p < 0.0001$ using one-way ANOVA followed by Dunnett's (A) or Šidák multiple comparison test (C); black asterisks indicate comparison to DMSO-treated siSCR control and blue to indicated VPS34i-treated siSCR control.

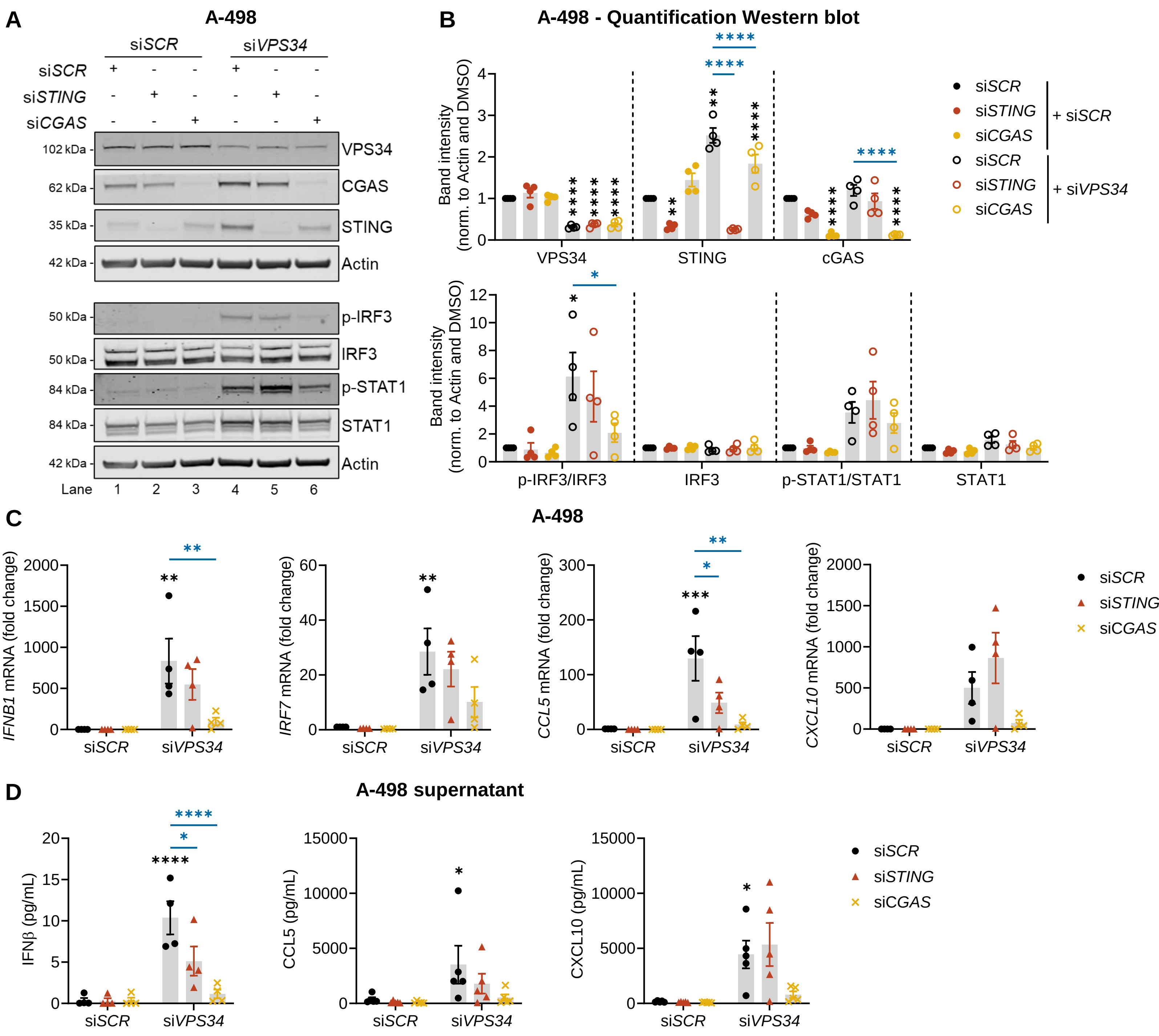

Supplementary Figure 3. Knockdown of VPS34 phenocopies increased type I IFN signaling and its cGAS/STING-dependency

(A-D) A-498 cells were reversely transfected with scrambled siRNA control (si*SCR*) or siRNA targeting STING (si*STING*) or cGAS (si*CGAS*). After 24h, cells were forwardly transfected with si*SCR* control or siRNA targeting VPS34 (si*VPS34*) for 48h. **(A)** Western blot images and **(B)** band intensity quantification of indicated proteins. Upper and lower blot were derived from the same lysate and performed simultaneously. **(C)** Quantification of *IFNB1*, *IRF7*, *CCL5*, and *CXCL10* gene expression using qRT-PCR and **(D)** IFNβ, CCL5, and CXCL10 protein in media supernatant using Mesoscale Discovery assays. Bars represent mean ± SEM of indicated sample numbers. * $p < 0.05$; ** $p < 0.01$; *** $p < 0.001$; **** $p < 0.0001$ using one-way ANOVA. Black asterisks indicate comparison to si*SCR*-treated control and blue asterisks specify comparison to si*SCR* + si*VPS34* control by Šidák multiple comparison test.

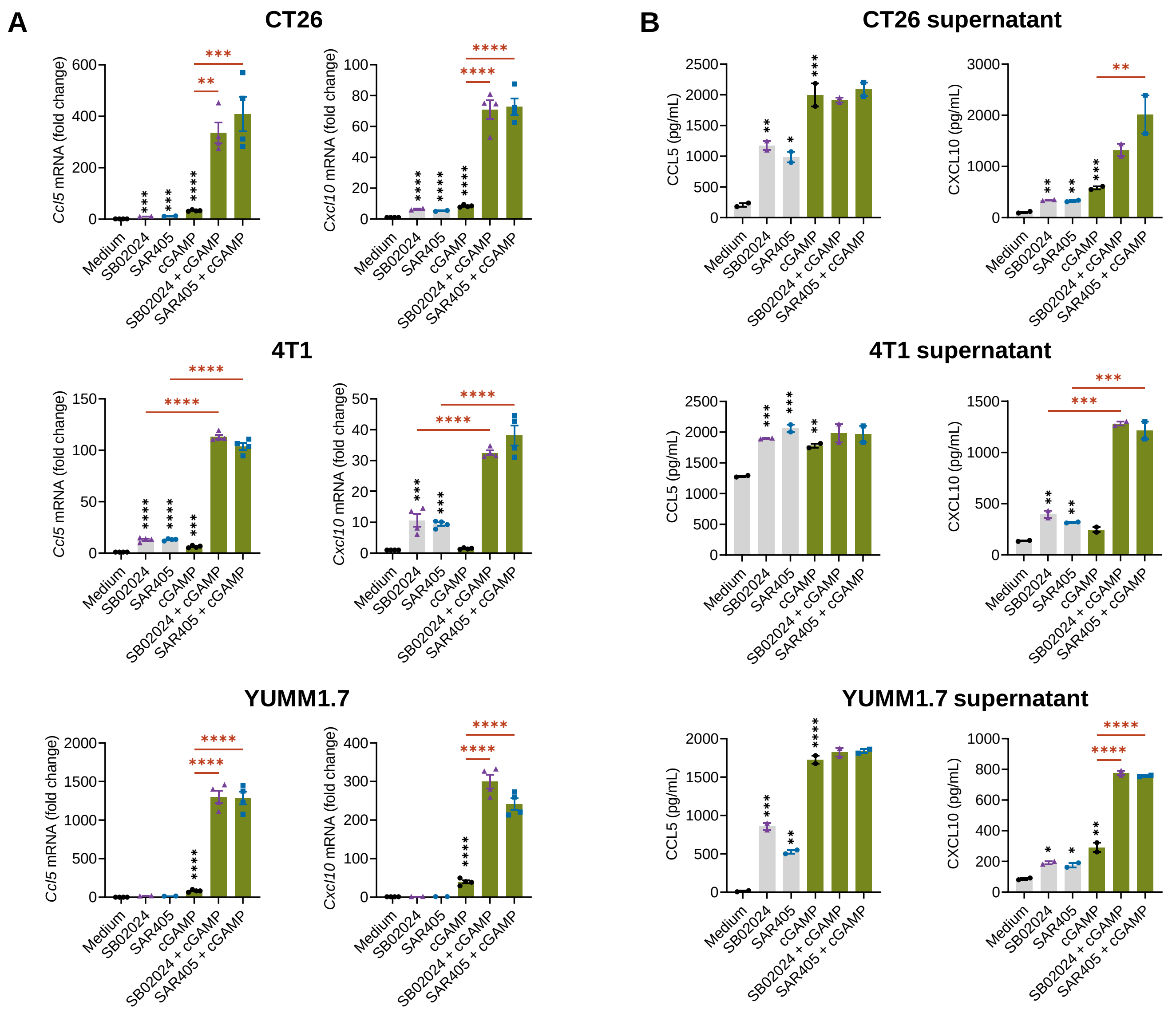

Supplementary Figure 4. VPS34i synergize with STING ligand cGAMP *in vitro*

(A, B) Murine cancer cell lines CT26 (colorectal cancer), 4T1 (triple-negative breast cancer), and YUMM1.7 (GEMM-derived melanoma) were treated with DMSO or VPS34i (5 µM SB02024 or 10 µM SAR405) in combination with 10 µg/mL cGAMP (green bars) for 24h. **(A)** Quantification of *Ccl5* and *Cxcl10* expression using qRT-PCR and **(B)** CCL5 and CXCL10 protein in the media supernatant using ELISA. Bars represent mean ± SEM of indicated sample numbers. * $p < 0.05$; ** $p < 0.01$; *** $p < 0.001$; **** $p < 0.0001$ using one-way ANOVA followed by Dunnett's (black asterisks indicating comparison to DMSO-treated control) or Šidák multiple comparison test (red asterisks indicating comparison of the combination treatment to the more effective single treatment).

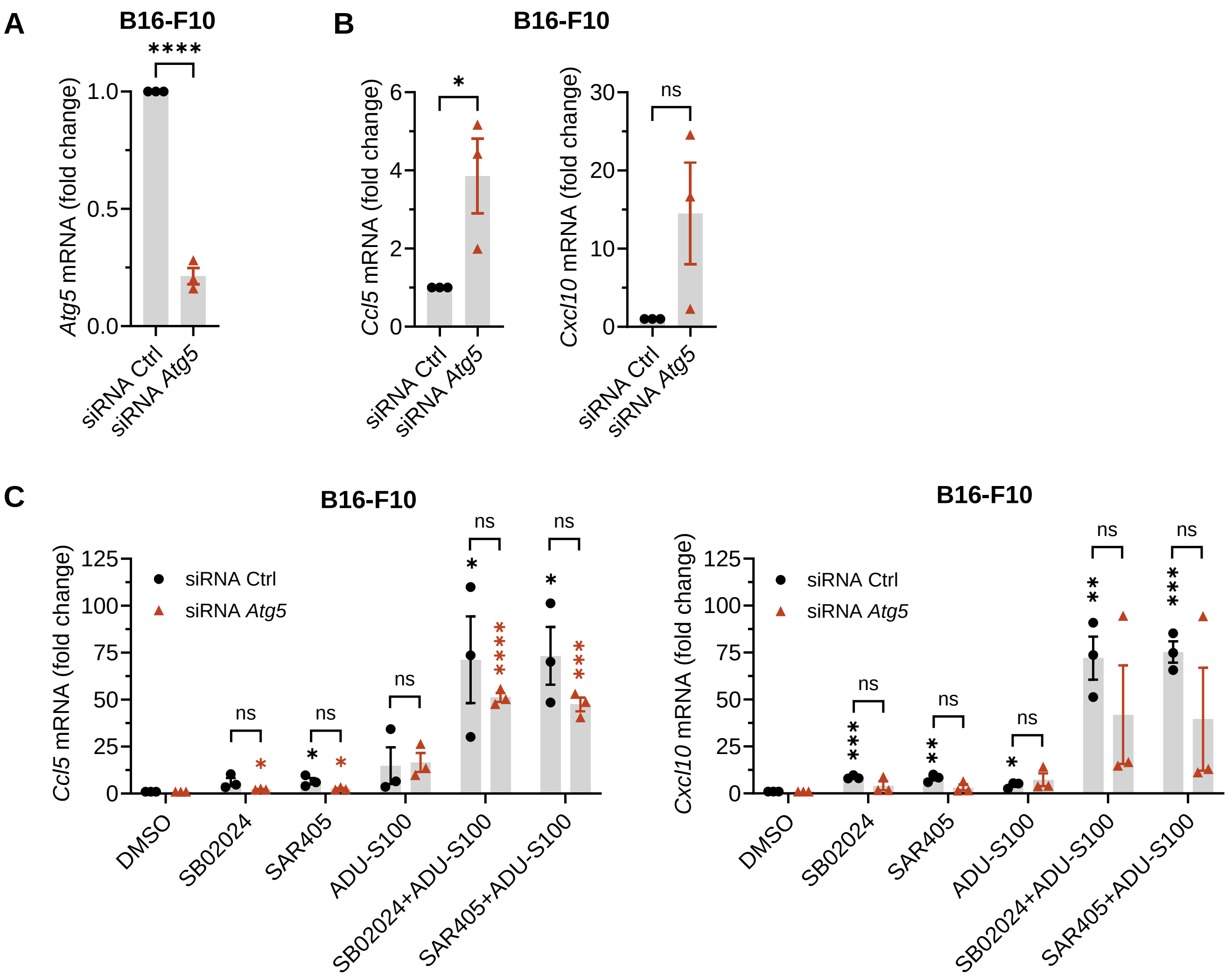

Supplementary Figure 5. Proinflammatory cytokine response induced by VPS34 inhibitor/ADU-S100 combination treatment is also observed in the background of *Atg5* knockdown.

(A, B) mRNA expression levels of *Atg5* **(A)**, *Ccl5*, and *Cxcl10* **(B)** depicted as fold changes compared to the control, observed in B16-F10 cells subjected to transfection with either control (Ctrl) siRNA or *Atg5* siRNA. **(C)** mRNA expression of *Ccl5* (left panel) and *Cxcl10* (right panel) in B16-F10 cells transfected with Ctrl or *Atg5* siRNA and treated for 24h with DMSO, 2 µM VPS34i (SB02024 or SAR405), or 5 µM ADU-S100 either individually or in combination. Data are reported as fold changes to each DMSO-treated control and represent mean ± SEM of three independent experiments. * $p < 0.05$; ** $p < 0.01$; *** $p < 0.001$; **** $p < 0.0001$ and ns: not significant, using unpaired two-tailed *t*-test. Single treatments were compared to the DMSO control and combination treatments were compared to the corresponding single treatments.

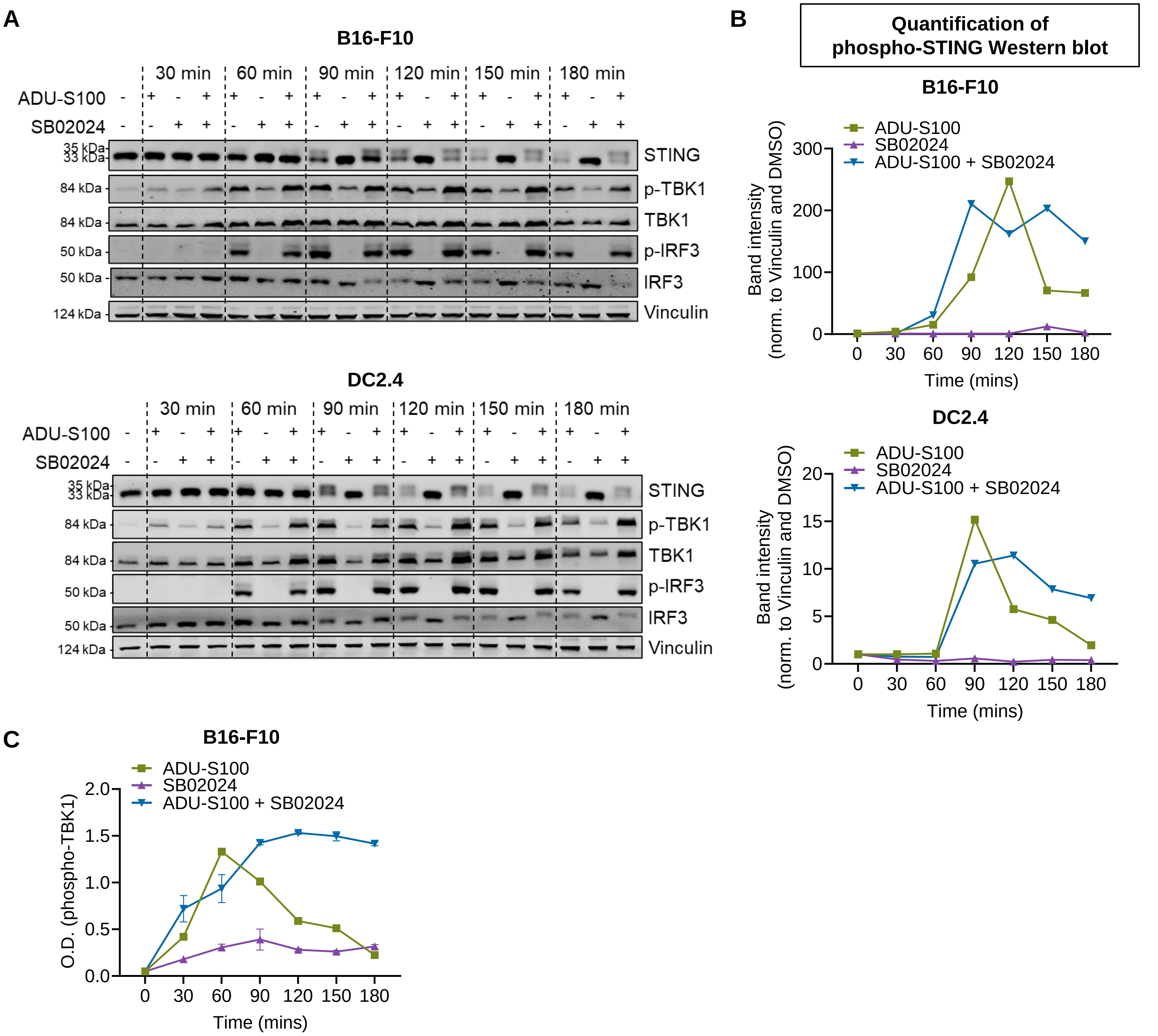

Supplementary Figure 6. VPS34 inhibitor SB02024 combined with STING agonist ADU-S100 induces prolonged activation of cGAS-STING pathway.

(A-C) B16-F10 **(A-C)** or DC2.4 **(A, B)** cells were treated with DMSO or 2 µM SB02024 in combination with 10 µg/mL ADU-S100 for indicated times. **(A)** Western blot images of indicated proteins are representative of two independent experiments. **(B)** Quantification of phospho-STING levels (upper STING band, known to be the activated form) in panel A. **(C)** Quantification of phospho-TBK1 levels in B16-F10 cell lysates using ELISA kit (RayBiotech, #PEL-TBK1-S172-1) according to manufacturer's instructions. Data shows mean ± SD of two independent experiments.

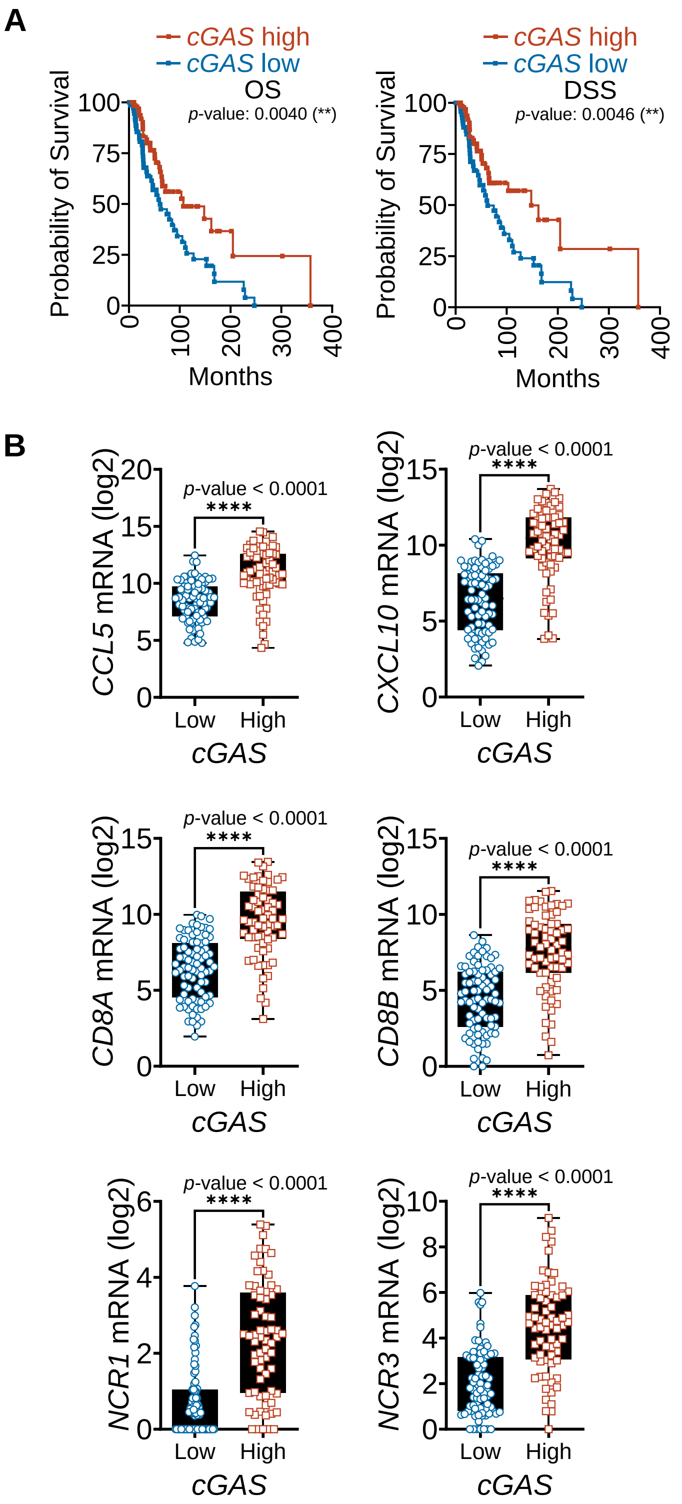

Supplementary Figure 7. Assessing the value of activating STING pathway in melanoma patients

(A) Kaplan-Meier overall survival (OS, left panels) and disease-specific survival (DSS, right panels) curves of melanoma patients expressing high and low mRNA of *cGAS*. Patients displaying high *cGAS* expression have significantly improved OS and DSS compared to those with low *cGAS* expression. The *p*-value of each curve was determined using the log-rank (Mantel-Cox) test. **(B)** mRNA expression levels of *CCL5* and *CXCL10* (upper panels), CD8 markers *CD8A* and *CD8B* (middle panels), and NK markers *NCR1* and *NCR3* (lower panels) in patients expressing high and low mRNA of *cGAS*. Statistically significant differences in high *cGAS* patients are calculated compared to low *cGAS* patients using an unpaired two-tailed *t*-test (**** $p<0.0001$).
