# Supplementary material for: Combining VPS34 inhibitors with STING agonists enhances type I interferon signaling and anti‐tumor efficacy
Source: Mol Oncol. 2024 Mar 20;18(8):1904–22. doi: 10.1002/1878-0261.13619 (PMC11306511; doi:10.1002/1878-0261.13619)
Supplement: Supplementary file 2 — Table S1. Data collection and refinement statistics for crystal structure. Table S2. Network enrichment analysis (NEA) network generation. Table S3. List of used siRNAs, antibodies, and RT‐qPCR primer sequences. Table S4. Overall survival (OS) information of the melanoma patients expressing high and low cGAS. Table S5. Disease‐specific survival (DSS) information of the melanoma patients expressing high and low cGAS. Table S6. Expression values of CCL5, CXCL10, CD8A, CD8B, NCR1, and NCR3 of the melanoma patients expressing high and low cGAS. [file MOL2-18-1904-s001.docx]

**Supplementary Tables**

**Supplementary Table 1**. **Data collection and refinement statistics for crystal structure** Diffraction data were collected at ESRF beamline ID29. Data were integrated and scaled using XDS [1]. Merging and truncation were performed with the CCP4 suite of programs [2]. An initial set of phases was determined by molecular replacement in Phaser [2] using PDB [3] entry 3LS8 [4] as a probe. The asymmetric unit contained two VPS34ΔC2 monomers. SB02024 was placed manually in the difference Fourier electron density maps. Final model was obtained following iterative cycles of manual model-building in Coot [5] and maximum-likelihood refinement in Refmac5 [6].

|  | **VPS34ΔC2/SB02024 complex** |
| --- | --- |
| PDB code | 8RXR |
| **Data collection** | |
| Beamline | ID29 |
| Wavelength (Å) | 0.97802 |
| Space group | C2 |
| **Cell dimensions** |  |
| *a*, *b*, *c (*Å) | 288.82, 98.65, 62.90 |
| *α*=*γ, β (*°) | 90, 92.27 |
| Resolution, Å | 49.33-2.06 (2.13-2.06)^a^ |
| *R_meas_* | 0.067 (1.150)^a^ |
| Mn*(I/sd)* | 12.6 (1.4)^a^ |
| *CC_1/2_* | 0.999 (0.530)^a^ |
| Completeness, % | 99.6 (99.3)^a^ |
| Multiplicity | 4.5 (4.4)^a^ |
| **Refinement** | |
| Resolution, Å | 49.37-2.06 |
| Number of reflections | 103076 |
| *R_work_/R_free_*^b^ | 0.18161/0.22450 |
| **Number of atoms** |  |
| Proteins | 8768 |
| Ligand (SB02024) | 89 |
| Water | 590 |
| Others (PEG, glycerol, DMSO, imidazole) | 57 |
| ***B*-factors, Å^2^** |  |
| Proteins | 47.64 |
| Ligand (SB02024) | 37.17 |
| Water | 51.13 |
| Others (PEG, glycerol, DMSO, imidazole) | 83.40 |
| **R.m.s. deviations** |  |
| Bond lengths, Å | 0.004 |
| Bond angles, ° | 1.315 |
| **Ramachandran analysis**^c^ | |
| Favored | 98.8% |
| Outliers | 0.0% |

^a^Values in parentheses are for the highest resolution shell.

^b^A set of 5% of reflections was used for cross-validation.

^c^Values determined by MolProbity[7]

*CC_1/2_*[8], percentage of correlation between intensities from random half-dataset; Mn, Mean; PDB, Protein Data Bank; R.m.s., root-mean-square; R_meas_[9]_._, redundancy independent R-factor.

**Supplementary Table 2. Network enrichment analysis (NEA) network generation**

NEA is a gene set enrichment analysis method detecting enrichment between a functional gene set (FGS: pathway, gene signature) and an altered gene set (AGS: a novel gene list characterizing an experimental state). The network space is searched for network edges connecting genes of AGS with any genes of FGS. The network was obtained by merging all edges of the mouse network predicted in FunCoup framework via Bayesian data integration [10] with all edges from curated databases. The resulting union network had 1,170,433 edges (functional coupling links) between 18,649 distinct MGI gene symbols. The collection included 611 FGSs. Each vehicle or SB02024-treated tumor sample was characterized by 30 genes (ASGs), expression of which was most upregulated compared to the rest of the sample collection using function samples2ags with options method=”topnorm” and Ntop=30 in the R package NEArender [11]. Significance was evaluated by matching the observed number of network edges between AGS and FGS to the number expected by chance for gene sets of the same node degrees and then calculating enrichment statistics, *p*-values, and *q*-values (or false discovery rate). Heatmaps were generated using Graphpad Prism.

| **Curated databases (source link)** |
| --- |
| KEGG (www.kegg.jp) |
| Phosphosite (www.phosphosite.org) |
| CORUM (www.mips.helmholtz-muenchen.de/corum/) |
| Innatedb (www.innatedb.com) |
| BioGrid (www.thebiogrid.org) |
| I2D (www.ophid.utoronto.ca/ophidv2.204) |
| **Functional gene set (source link)** |
| 53 NanoString signatures: 25 functional, 14 cell type markers, 14 annotation sets (www.nanostring.com) |
| 326 KEGG mouse pathways (www.kegg.jp) |
| 179 WikiPathways (www.wikipathways.org) |
| 26 relevant GO terms (www.geneontology.org) |
| 2 relevant Reactome pathways (www.reactome.org) |
| 25 custom gene sets derived from literature [12–19] |

**Supplementary Table 3. List of used siRNAs, antibodies, and RT-qPCR primer sequences**

| **siRNA** | **Cat. number** | **Supplier** |
| --- | --- | --- |
| non-targeting pool si*Scramble* (si*SCR*) | D-001810-10-05,  ONTARGET*plus* SMARTPool | Dharmacon |
| human *TMEM173* siRNA (si*STING)* | L-024333-00-0005,  ONTARGET*plus* SMARTPool | Dharmacon |
| human *MB21D1* siRNA (si*CGAS)* | L-015607-02-0005,  ONTARGET*plus* SMARTPool | Dharmacon |
| human *TBK1* siRNA (si*TBK1)* | L-003788-00-0005,  ONTARGET*plus* SMARTPool | Dharmacon |
| human *PIK3C3* siRNA (si*VPS34*) | L-005250-00-0005,  ONTARGET*plus* SMARTPool | Dharmacon |
| mouse *Atg5* siRNA (si*Atg5*) | Mm_Apg5l_5 FlexiTube siRNA | Qiagen |

| **Antibody** | **Dilution** | **Diluent** | **Cat. number** | **Supplier** |
| --- | --- | --- | --- | --- |
| STING | 1:1000 | 5% milk in TBS-T | 13647 | Cell Signaling Technology |
| cGAS | 1:1000 | 5% milk in TBS-T | 15102 | Cell Signaling Technology |
| TBK1 | 1:1000 | 5% milk in TBS-T | 3504 | Cell Signaling Technology |
| phospho-TBK1 Ser172 | 1:1000 | 5% BSA in TBS-T | 5483 | Cell Signaling Technology |
| IRF3 | 1:1000 | 5% milk in TBS-T | 4302 | Cell Signaling Technology |
| phospho-IRF3 Ser386 | 1:500 | 5% BSA in TBS-T | 37829 | Cell Signaling Technology |
| STAT1 | 1:1000 | 5% milk in TBS-T | 9172 | Cell Signaling Technology |
| phospho-STAT1 Tyr701 | 1:1000 | 5% BSA in TBS-T | 7649 | Cell Signaling Technology |
| VPS34 | 1:1000 | 5% milk in TBS-T | GTX129528 | GeneTex |
| Actin | 1:10,000 | 5% milk in TBS-T | A5441 | Sigma-Aldrich |
| Vinculin | 1:3000 | 5% milk in TBS-T | ab129002 | Abcam |
| IRDye 800CW  goat anti-rabbit IgG (H+L) | 1:10000 | Blocking buffer  (#927-60001, LI-COR) | 926-32211 | LI-COR |
| IRDye 680RD  donkey anti-mouse IgG (H+L) | 1:10000 | Blocking buffer  (#927-60001, LI-COR) | 926-68072 | LI-COR |

| **Human primers** | **Forward primer sequence** | **Reverse primer sequence** |
| --- | --- | --- |
| *IFNB1* | GCTTGGATTCCTACAAAGAAGCA | ATAGATGGTCAATGCGGCGTC |
| *IRF1* | CTGTGCGAGTGTACCGGATG | ATCCCCACATGACTTCCTCTT |
| *IRF7* | GCTGGACGTGACCATCATGTA | GGGCCGTATAGGAACGTGC |
| *IRF9* | GCCCTACAAGGTGTATCAGTTG | TGCTGTCGCTTTGATGGTACT |
| *CCL5* | CTCCCCATATTCCTCGGACA | ACTCCTTGATGTGGGCACG |
| *CXCL10* | TGGCATTCAAGGAGTACCTCTC | ATGCTGATGCAGGTACAGCG |
| *Tubulin*  (housekeeping gene) | GAAGCAGCAACCATGCGTGA | GGCATTGCCAATCTGGACAC |
| **Mouse primers** | **Forward primer sequence** | **Reverse primer sequence** |
| *Ccl5* | GCTGCTTTGCCTACCTCTCC | TCGAGTGACAAACACGACTGC |
| *Cxcl10* | CCAAGTGCTGCCGTCATTTTC | GGCTCGCAGGGATGATTTCAA |
| *Ifnb1* | CAGCTCCAAGAAAGGACGAAC | GGCAGTGTAACTCTTCTGCAT |
| *Sting1* | GGTCACCGCTCCAAATATGTAG | CAGTAGTCCAAGTTCGTGCGA |
| *Atg5* | CTTGCATCAAGTTCAGCTCTTCC | AAGTGAGCCTCAACCGCATCCT |
| *Rps18*  (housekeeping gene) | AGTTCCAGCACATTTTGCGAG | TCATCCTCCGTGAGTTCTCCA |

**Supplementary Table 4. Overall survival (OS) information of the melanoma patients expressing high and low *cGAS***

| **Overall survival** | | | | | | |
| --- | --- | --- | --- | --- | --- | --- |
| **(A) cGAS High** | | | | | | |
| **Case ID** | **Study ID** | **Number at Risk** | **Status** | | **Survival Rate** | **Time (months)** |
| TCGA-EB-A5VV | skcm_tcga_pan_can_atlas_2018 | 66 | censored | 0 | 1.00 | 7.04 |
| TCGA-GF-A3OT | skcm_tcga_pan_can_atlas_2018 | 65 | censored | 0 | 1.00 | 9.90 |
| TCGA-GN-A266 | skcm_tcga_pan_can_atlas_2018 | 64 | deceased | 1 | 0.98 | 10.13 |
| TCGA-EB-A6QY | skcm_tcga_pan_can_atlas_2018 | 63 | censored | 0 | 0.98 | 12.56 |
| TCGA-EE-A29N | skcm_tcga_pan_can_atlas_2018 | 62 | deceased | 1 | 0.97 | 18.61 |
| TCGA-FR-A7U9 | skcm_tcga_pan_can_atlas_2018 | 61 | censored | 0 | 0.97 | 18.77 |
| TCGA-GF-A4EO | skcm_tcga_pan_can_atlas_2018 | 60 | censored | 0 | 0.97 | 19.43 |
| TCGA-EE-A2M8 | skcm_tcga_pan_can_atlas_2018 | 59 | deceased | 1 | 0.95 | 19.76 |
| TCGA-EE-A2M5 | skcm_tcga_pan_can_atlas_2018 | 58 | deceased | 1 | 0.94 | 21.67 |
| TCGA-D3-A51N | skcm_tcga_pan_can_atlas_2018 | 57 | censored | 0 | 0.94 | 22.62 |
| TCGA-D3-A2J9 | skcm_tcga_pan_can_atlas_2018 | 56 | deceased | 1 | 0.92 | 23.77 |
| TCGA-GN-A26C | skcm_tcga_pan_can_atlas_2018 | 55 | deceased | 1 | 0.90 | 26.99 |
| TCGA-DA-A1IB | skcm_tcga_pan_can_atlas_2018 | 54 | censored | 0 | 0.90 | 27.12 |
| TCGA-EE-A17Y | skcm_tcga_pan_can_atlas_2018 | 53 | deceased | 1 | 0.89 | 27.22 |
| TCGA-EE-A3JD | skcm_tcga_pan_can_atlas_2018 | 52 | deceased | 1 | 0.87 | 27.35 |
| TCGA-FS-A1Z4 | skcm_tcga_pan_can_atlas_2018 | 51 | deceased | 1 | 0.85 | 28.08 |
| TCGA-D3-A1Q8 | skcm_tcga_pan_can_atlas_2018 | 50 | deceased | 1 | 0.83 | 28.08 |
| TCGA-GF-A769 | skcm_tcga_pan_can_atlas_2018 | 49 | deceased | 1 | 0.82 | 35.18 |
| TCGA-ER-A195 | skcm_tcga_pan_can_atlas_2018 | 48 | deceased | 1 | 0.80 | 35.44 |
| TCGA-FR-A7UA | skcm_tcga_pan_can_atlas_2018 | 47 | censored | 0 | 0.80 | 38.27 |
| TCGA-ER-A2NH | skcm_tcga_pan_can_atlas_2018 | 46 | censored | 0 | 0.80 | 41.56 |
| TCGA-EE-A3AG | skcm_tcga_pan_can_atlas_2018 | 45 | deceased | 1 | 0.78 | 41.59 |
| TCGA-W3-AA1V | skcm_tcga_pan_can_atlas_2018 | 44 | deceased | 1 | 0.76 | 42.08 |
| TCGA-DA-A3F8 | skcm_tcga_pan_can_atlas_2018 | 43 | censored | 0 | 0.76 | 43.36 |
| TCGA-D3-A3C8 | skcm_tcga_pan_can_atlas_2018 | 42 | censored | 0 | 0.76 | 46.32 |
| TCGA-YD-A9TA | skcm_tcga_pan_can_atlas_2018 | 41 | censored | 0 | 0.76 | 49.18 |
| TCGA-ER-A19S | skcm_tcga_pan_can_atlas_2018 | 40 | censored | 0 | 0.76 | 49.48 |
| TCGA-FS-A1ZW | skcm_tcga_pan_can_atlas_2018 | 39 | censored | 0 | 0.76 | 49.48 |
| TCGA-ER-A198 | skcm_tcga_pan_can_atlas_2018 | 38 | deceased | 1 | 0.74 | 50.76 |
| TCGA-ER-A19Q | skcm_tcga_pan_can_atlas_2018 | 37 | deceased | 1 | 0.72 | 50.89 |
| TCGA-EE-A3JE | skcm_tcga_pan_can_atlas_2018 | 36 | censored | 0 | 0.72 | 51.35 |
| TCGA-FS-A1ZD | skcm_tcga_pan_can_atlas_2018 | 35 | deceased | 1 | 0.70 | 53.52 |
| TCGA-EE-A2GK | skcm_tcga_pan_can_atlas_2018 | 34 | censored | 0 | 0.70 | 54.74 |
| TCGA-D3-A51F | skcm_tcga_pan_can_atlas_2018 | 33 | censored | 0 | 0.70 | 55.73 |
| TCGA-D3-A51H | skcm_tcga_pan_can_atlas_2018 | 32 | censored | 0 | 0.70 | 56.35 |
| TCGA-D3-A3CE | skcm_tcga_pan_can_atlas_2018 | 31 | deceased | 1 | 0.68 | 60.23 |
| TCGA-EE-A2MC | skcm_tcga_pan_can_atlas_2018 | 30 | deceased | 1 | 0.66 | 61.51 |
| TCGA-D3-A2JF | skcm_tcga_pan_can_atlas_2018 | 29 | censored | 0 | 0.66 | 62.07 |
| TCGA-GN-A267 | skcm_tcga_pan_can_atlas_2018 | 28 | deceased | 1 | 0.63 | 64.44 |
| TCGA-D3-A2J8 | skcm_tcga_pan_can_atlas_2018 | 27 | deceased | 1 | 0.61 | 65.49 |
| TCGA-HR-A2OH | skcm_tcga_pan_can_atlas_2018 | 26 | deceased | 1 | 0.59 | 65.88 |
| TCGA-D3-A2JO | skcm_tcga_pan_can_atlas_2018 | 25 | censored | 0 | 0.59 | 66.08 |
| TCGA-EB-A5SG | skcm_tcga_pan_can_atlas_2018 | 24 | censored | 0 | 0.59 | 68.25 |
| TCGA-EE-A2MT | skcm_tcga_pan_can_atlas_2018 | 23 | censored | 0 | 0.59 | 71.21 |
| TCGA-D3-A1Q6 | skcm_tcga_pan_can_atlas_2018 | 22 | deceased | 1 | 0.56 | 71.80 |
| TCGA-DA-A95X | skcm_tcga_pan_can_atlas_2018 | 21 | censored | 0 | 0.56 | 73.94 |
| TCGA-DA-A1HV | skcm_tcga_pan_can_atlas_2018 | 20 | censored | 0 | 0.56 | 76.57 |
| TCGA-ER-A19A | skcm_tcga_pan_can_atlas_2018 | 19 | censored | 0 | 0.56 | 77.75 |
| TCGA-D3-A2JC | skcm_tcga_pan_can_atlas_2018 | 18 | censored | 0 | 0.56 | 86.76 |
| TCGA-GN-A265 | skcm_tcga_pan_can_atlas_2018 | 17 | censored | 0 | 0.56 | 96.92 |
| TCGA-EE-A2ME | skcm_tcga_pan_can_atlas_2018 | 16 | deceased | 1 | 0.53 | 103.26 |
| TCGA-ER-A1A1 | skcm_tcga_pan_can_atlas_2018 | 15 | censored | 0 | 0.53 | 105.07 |
| TCGA-D3-A8GM | skcm_tcga_pan_can_atlas_2018 | 14 | deceased | 1 | 0.49 | 107.14 |
| TCGA-D3-A2JA | skcm_tcga_pan_can_atlas_2018 | 13 | censored | 0 | 0.49 | 115.53 |
| TCGA-EE-A3AB | skcm_tcga_pan_can_atlas_2018 | 12 | censored | 0 | 0.49 | 122.73 |
| TCGA-D3-A1Q7 | skcm_tcga_pan_can_atlas_2018 | 11 | censored | 0 | 0.49 | 133.25 |
| TCGA-EE-A2MR | skcm_tcga_pan_can_atlas_2018 | 10 | censored | 0 | 0.49 | 134.40 |
| TCGA-D3-A5GN | skcm_tcga_pan_can_atlas_2018 | 9 | censored | 0 | 0.49 | 135.75 |
| TCGA-ER-A19W | skcm_tcga_pan_can_atlas_2018 | 8 | deceased | 1 | 0.43 | 148.17 |
| TCGA-ER-A19P | skcm_tcga_pan_can_atlas_2018 | 7 | deceased | 1 | 0.37 | 162.08 |
| TCGA-FR-A44A | skcm_tcga_pan_can_atlas_2018 | 6 | censored | 0 | 0.37 | 174.21 |
| TCGA-D3-A5GR | skcm_tcga_pan_can_atlas_2018 | 5 | censored | 0 | 0.37 | 178.32 |
| TCGA-EE-A3JB | skcm_tcga_pan_can_atlas_2018 | 4 | censored | 0 | 0.37 | 201.80 |
| TCGA-EE-A2MI | skcm_tcga_pan_can_atlas_2018 | 3 | deceased | 1 | 0.24 | 204.66 |
| TCGA-ER-A19G | skcm_tcga_pan_can_atlas_2018 | 2 | censored | 0 | 0.24 | 302.07 |
| TCGA-FS-A1ZC | skcm_tcga_pan_can_atlas_2018 | 1 | deceased | 1 | 0.00 | 357.37 |
|  |  |  |  |  |  |  |
| **(B) cGAS Low** | | | | | | |
| **Case ID** | **Study ID** | **Number at Risk** | **Status** | | **Survival Rate** | **Time (months)** |
| TCGA-GF-A2C7 | skcm_tcga_pan_can_atlas_2018 | 77 | censored | 0 | 1.00 | 0.69 |
| TCGA-EE-A29L | skcm_tcga_pan_can_atlas_2018 | 76 | deceased | 1 | 0.99 | 2.60 |
| TCGA-EB-A3XE | skcm_tcga_pan_can_atlas_2018 | 75 | censored | 0 | 0.99 | 5.92 |
| TCGA-YD-A89C | skcm_tcga_pan_can_atlas_2018 | 74 | censored | 0 | 0.99 | 6.90 |
| TCGA-QB-A6FS | skcm_tcga_pan_can_atlas_2018 | 73 | censored | 0 | 0.99 | 7.23 |
| TCGA-EB-A553 | skcm_tcga_pan_can_atlas_2018 | 72 | censored | 0 | 0.99 | 7.43 |
| TCGA-EB-A41A | skcm_tcga_pan_can_atlas_2018 | 71 | censored | 0 | 0.99 | 8.61 |
| TCGA-ER-A19T | skcm_tcga_pan_can_atlas_2018 | 70 | deceased | 1 | 0.97 | 8.88 |
| TCGA-FS-A1ZG | skcm_tcga_pan_can_atlas_2018 | 69 | deceased | 1 | 0.96 | 9.70 |
| TCGA-GN-A4U7 | skcm_tcga_pan_can_atlas_2018 | 68 | deceased | 1 | 0.94 | 10.42 |
| TCGA-BF-A5EQ | skcm_tcga_pan_can_atlas_2018 | 67 | censored | 0 | 0.94 | 10.62 |
| TCGA-BF-A5ER | skcm_tcga_pan_can_atlas_2018 | 66 | censored | 0 | 0.94 | 10.75 |
| TCGA-D3-A2JK | skcm_tcga_pan_can_atlas_2018 | 65 | deceased | 1 | 0.93 | 12.10 |
| TCGA-EB-A5SF | skcm_tcga_pan_can_atlas_2018 | 64 | deceased | 1 | 0.92 | 12.13 |
| TCGA-DA-A95Z | skcm_tcga_pan_can_atlas_2018 | 63 | censored | 0 | 0.92 | 13.02 |
| TCGA-EB-A5SE | skcm_tcga_pan_can_atlas_2018 | 62 | deceased | 1 | 0.90 | 13.18 |
| TCGA-XV-AB01 | skcm_tcga_pan_can_atlas_2018 | 61 | censored | 0 | 0.90 | 13.25 |
| TCGA-D3-A3ML | skcm_tcga_pan_can_atlas_2018 | 60 | deceased | 1 | 0.89 | 13.87 |
| TCGA-YG-AA3P | skcm_tcga_pan_can_atlas_2018 | 59 | censored | 0 | 0.89 | 14.43 |
| TCGA-EE-A182 | skcm_tcga_pan_can_atlas_2018 | 58 | deceased | 1 | 0.87 | 14.70 |
| TCGA-ER-A19K | skcm_tcga_pan_can_atlas_2018 | 57 | deceased | 1 | 0.86 | 15.42 |
| TCGA-D3-A5GT | skcm_tcga_pan_can_atlas_2018 | 56 | censored | 0 | 0.86 | 16.01 |
| TCGA-BF-A5ES | skcm_tcga_pan_can_atlas_2018 | 55 | censored | 0 | 0.86 | 16.11 |
| TCGA-IH-A3EA | skcm_tcga_pan_can_atlas_2018 | 54 | censored | 0 | 0.86 | 17.23 |
| TCGA-EB-A5KH | skcm_tcga_pan_can_atlas_2018 | 53 | deceased | 1 | 0.84 | 20.35 |
| TCGA-DA-A1I0 | skcm_tcga_pan_can_atlas_2018 | 52 | deceased | 1 | 0.82 | 20.38 |
| TCGA-GN-A8LL | skcm_tcga_pan_can_atlas_2018 | 51 | deceased | 1 | 0.81 | 21.37 |
| TCGA-WE-AAA3 | skcm_tcga_pan_can_atlas_2018 | 50 | censored | 0 | 0.81 | 21.40 |
| TCGA-FR-A3R1 | skcm_tcga_pan_can_atlas_2018 | 49 | censored | 0 | 0.81 | 22.52 |
| TCGA-BF-A3DL | skcm_tcga_pan_can_atlas_2018 | 48 | censored | 0 | 0.81 | 25.28 |
| TCGA-EB-A5UM | skcm_tcga_pan_can_atlas_2018 | 47 | censored | 0 | 0.81 | 25.61 |
| TCGA-EE-A29V | skcm_tcga_pan_can_atlas_2018 | 46 | deceased | 1 | 0.79 | 25.87 |
| TCGA-D3-A51T | skcm_tcga_pan_can_atlas_2018 | 45 | censored | 0 | 0.79 | 26.89 |
| TCGA-FS-A1ZZ | skcm_tcga_pan_can_atlas_2018 | 44 | deceased | 1 | 0.77 | 27.02 |
| TCGA-FS-A1ZY | skcm_tcga_pan_can_atlas_2018 | 43 | deceased | 1 | 0.75 | 27.09 |
| TCGA-BF-A1Q0 | skcm_tcga_pan_can_atlas_2018 | 42 | censored | 0 | 0.75 | 27.32 |
| TCGA-D3-A2JE | skcm_tcga_pan_can_atlas_2018 | 41 | deceased | 1 | 0.73 | 27.65 |
| TCGA-FR-A7U8 | skcm_tcga_pan_can_atlas_2018 | 40 | censored | 0 | 0.73 | 27.85 |
| TCGA-ER-A2NB | skcm_tcga_pan_can_atlas_2018 | 39 | deceased | 1 | 0.72 | 28.18 |
| TCGA-FS-A4F5 | skcm_tcga_pan_can_atlas_2018 | 38 | deceased | 1 | 0.70 | 28.73 |
| TCGA-ER-A2NF | skcm_tcga_pan_can_atlas_2018 | 37 | deceased | 1 | 0.68 | 28.83 |
| TCGA-EB-A4OY | skcm_tcga_pan_can_atlas_2018 | 36 | censored | 0 | 0.68 | 32.12 |
| TCGA-D3-A51K | skcm_tcga_pan_can_atlas_2018 | 35 | censored | 0 | 0.68 | 32.94 |
| TCGA-FS-A4F9 | skcm_tcga_pan_can_atlas_2018 | 34 | censored | 0 | 0.68 | 34.03 |
| TCGA-FR-A8YC | skcm_tcga_pan_can_atlas_2018 | 33 | deceased | 1 | 0.66 | 34.82 |
| TCGA-FR-A8YD | skcm_tcga_pan_can_atlas_2018 | 32 | deceased | 1 | 0.64 | 36.26 |
| TCGA-DA-A95W | skcm_tcga_pan_can_atlas_2018 | 31 | censored | 0 | 0.64 | 37.35 |
| TCGA-EB-A3XD | skcm_tcga_pan_can_atlas_2018 | 30 | censored | 0 | 0.64 | 38.14 |
| TCGA-GN-A4U4 | skcm_tcga_pan_can_atlas_2018 | 29 | censored | 0 | 0.64 | 39.35 |
| TCGA-ER-A194 | skcm_tcga_pan_can_atlas_2018 | 28 | deceased | 1 | 0.61 | 44.51 |
| TCGA-ER-A3EV | skcm_tcga_pan_can_atlas_2018 | 27 | deceased | 1 | 0.59 | 46.98 |
| TCGA-FS-A1ZJ | skcm_tcga_pan_can_atlas_2018 | 26 | deceased | 1 | 0.57 | 47.37 |
| TCGA-EB-A5SH | skcm_tcga_pan_can_atlas_2018 | 25 | censored | 0 | 0.57 | 54.02 |
| TCGA-FS-A4FC | skcm_tcga_pan_can_atlas_2018 | 24 | deceased | 1 | 0.55 | 54.41 |
| TCGA-D3-A8GI | skcm_tcga_pan_can_atlas_2018 | 23 | deceased | 1 | 0.52 | 58.52 |
| TCGA-EE-A29S | skcm_tcga_pan_can_atlas_2018 | 22 | deceased | 1 | 0.50 | 61.28 |
| TCGA-GN-A268 | skcm_tcga_pan_can_atlas_2018 | 21 | deceased | 1 | 0.47 | 62.79 |
| TCGA-FS-A1ZP | skcm_tcga_pan_can_atlas_2018 | 20 | deceased | 1 | 0.45 | 74.73 |
| TCGA-D9-A6EC | skcm_tcga_pan_can_atlas_2018 | 19 | censored | 0 | 0.45 | 77.56 |
| TCGA-FS-A4F0 | skcm_tcga_pan_can_atlas_2018 | 18 | censored | 0 | 0.45 | 77.82 |
| TCGA-D3-A8GC | skcm_tcga_pan_can_atlas_2018 | 17 | deceased | 1 | 0.42 | 79.59 |
| TCGA-EE-A29B | skcm_tcga_pan_can_atlas_2018 | 16 | deceased | 1 | 0.40 | 85.08 |
| TCGA-D3-A8GL | skcm_tcga_pan_can_atlas_2018 | 15 | deceased | 1 | 0.37 | 89.13 |
| TCGA-D3-A1QA | skcm_tcga_pan_can_atlas_2018 | 14 | censored | 0 | 0.37 | 90.90 |
| TCGA-EE-A180 | skcm_tcga_pan_can_atlas_2018 | 13 | deceased | 1 | 0.34 | 94.98 |
| TCGA-W3-AA21 | skcm_tcga_pan_can_atlas_2018 | 12 | deceased | 1 | 0.31 | 105.04 |
| TCGA-W3-AA1R | skcm_tcga_pan_can_atlas_2018 | 11 | deceased | 1 | 0.29 | 111.09 |
| TCGA-D3-A2JG | skcm_tcga_pan_can_atlas_2018 | 10 | deceased | 1 | 0.26 | 113.52 |
| TCGA-EE-A3J4 | skcm_tcga_pan_can_atlas_2018 | 9 | deceased | 1 | 0.23 | 127.20 |
| TCGA-D3-A8GP | skcm_tcga_pan_can_atlas_2018 | 8 | censored | 0 | 0.23 | 152.74 |
| TCGA-EE-A3JI | skcm_tcga_pan_can_atlas_2018 | 7 | deceased | 1 | 0.20 | 152.81 |
| TCGA-EE-A2MS | skcm_tcga_pan_can_atlas_2018 | 6 | censored | 0 | 0.20 | 162.47 |
| TCGA-EE-A2MM | skcm_tcga_pan_can_atlas_2018 | 5 | deceased | 1 | 0.16 | 167.90 |
| TCGA-EE-A20H | skcm_tcga_pan_can_atlas_2018 | 4 | deceased | 1 | 0.12 | 168.26 |
| TCGA-DA-A3F5 | skcm_tcga_pan_can_atlas_2018 | 3 | deceased | 1 | 0.08 | 225.96 |
| TCGA-FS-A1YY | skcm_tcga_pan_can_atlas_2018 | 2 | deceased | 1 | 0.04 | 228.59 |
| TCGA-ER-A3ES | skcm_tcga_pan_can_atlas_2018 | 1 | deceased | 1 | 0.00 | 247.03 |

**Supplementary Table 5. Disease-specific survival (DSS) information of the melanoma patients expressing high and low *cGAS***

| **Disease-specific survival (DSS)** | | | | | | |
| --- | --- | --- | --- | --- | --- | --- |
| **(A) cGAS High** | | | | | | |
| **Case ID** | **Study ID** | **Number at Risk** | **Status** | | **Survival Rate** | **Time (months)** |
| TCGA-EB-A5VV | skcm_tcga_pan_can_atlas_2018 | 66 | censored | 0 | 1.00 | 7.04 |
| TCGA-GF-A3OT | skcm_tcga_pan_can_atlas_2018 | 65 | censored | 0 | 1.00 | 9.90 |
| TCGA-GN-A266 | skcm_tcga_pan_can_atlas_2018 | 64 | deceased | 1 | 0.98 | 10.13 |
| TCGA-EB-A6QY | skcm_tcga_pan_can_atlas_2018 | 63 | censored | 0 | 0.98 | 12.56 |
| TCGA-EE-A29N | skcm_tcga_pan_can_atlas_2018 | 62 | deceased | 1 | 0.97 | 18.61 |
| TCGA-FR-A7U9 | skcm_tcga_pan_can_atlas_2018 | 61 | censored | 0 | 0.97 | 18.77 |
| TCGA-GF-A4EO | skcm_tcga_pan_can_atlas_2018 | 60 | censored | 0 | 0.97 | 19.43 |
| TCGA-EE-A2M8 | skcm_tcga_pan_can_atlas_2018 | 59 | deceased | 1 | 0.95 | 19.76 |
| TCGA-EE-A2M5 | skcm_tcga_pan_can_atlas_2018 | 58 | deceased | 1 | 0.94 | 21.67 |
| TCGA-D3-A51N | skcm_tcga_pan_can_atlas_2018 | 57 | censored | 0 | 0.94 | 22.62 |
| TCGA-D3-A2J9 | skcm_tcga_pan_can_atlas_2018 | 56 | deceased | 1 | 0.92 | 23.77 |
| TCGA-GN-A26C | skcm_tcga_pan_can_atlas_2018 | 55 | deceased | 1 | 0.90 | 26.99 |
| TCGA-DA-A1IB | skcm_tcga_pan_can_atlas_2018 | 54 | censored | 0 | 0.90 | 27.12 |
| TCGA-EE-A17Y | skcm_tcga_pan_can_atlas_2018 | 53 | deceased | 1 | 0.89 | 27.22 |
| TCGA-EE-A3JD | skcm_tcga_pan_can_atlas_2018 | 52 | deceased | 1 | 0.87 | 27.35 |
| TCGA-FS-A1Z4 | skcm_tcga_pan_can_atlas_2018 | 51 | deceased | 1 | 0.85 | 28.08 |
| TCGA-D3-A1Q8 | skcm_tcga_pan_can_atlas_2018 | 50 | deceased | 1 | 0.83 | 28.08 |
| TCGA-GF-A769 | skcm_tcga_pan_can_atlas_2018 | 49 | deceased | 1 | 0.82 | 35.18 |
| TCGA-ER-A195 | skcm_tcga_pan_can_atlas_2018 | 48 | deceased | 1 | 0.80 | 35.44 |
| TCGA-FR-A7UA | skcm_tcga_pan_can_atlas_2018 | 47 | censored | 0 | 0.80 | 38.27 |
| TCGA-ER-A2NH | skcm_tcga_pan_can_atlas_2018 | 46 | censored | 0 | 0.80 | 41.56 |
| TCGA-EE-A3AG | skcm_tcga_pan_can_atlas_2018 | 45 | deceased | 1 | 0.78 | 41.59 |
| TCGA-W3-AA1V | skcm_tcga_pan_can_atlas_2018 | 44 | deceased | 1 | 0.76 | 42.08 |
| TCGA-DA-A3F8 | skcm_tcga_pan_can_atlas_2018 | 43 | censored | 0 | 0.76 | 43.36 |
| TCGA-D3-A3C8 | skcm_tcga_pan_can_atlas_2018 | 42 | censored | 0 | 0.76 | 46.32 |
| TCGA-YD-A9TA | skcm_tcga_pan_can_atlas_2018 | 41 | censored | 0 | 0.76 | 49.18 |
| TCGA-ER-A19S | skcm_tcga_pan_can_atlas_2018 | 40 | censored | 0 | 0.76 | 49.48 |
| TCGA-FS-A1ZW | skcm_tcga_pan_can_atlas_2018 | 39 | censored | 0 | 0.76 | 49.48 |
| TCGA-ER-A198 | skcm_tcga_pan_can_atlas_2018 | 38 | deceased | 1 | 0.74 | 50.76 |
| TCGA-ER-A19Q | skcm_tcga_pan_can_atlas_2018 | 37 | deceased | 1 | 0.72 | 50.89 |
| TCGA-EE-A3JE | skcm_tcga_pan_can_atlas_2018 | 36 | censored | 0 | 0.72 | 51.35 |
| TCGA-FS-A1ZD | skcm_tcga_pan_can_atlas_2018 | 35 | deceased | 1 | 0.70 | 53.52 |
| TCGA-EE-A2GK | skcm_tcga_pan_can_atlas_2018 | 34 | censored | 0 | 0.70 | 54.74 |
| TCGA-D3-A51F | skcm_tcga_pan_can_atlas_2018 | 33 | censored | 0 | 0.70 | 55.73 |
| TCGA-D3-A51H | skcm_tcga_pan_can_atlas_2018 | 32 | censored | 0 | 0.70 | 56.35 |
| TCGA-D3-A3CE | skcm_tcga_pan_can_atlas_2018 | 31 | deceased | 1 | 0.68 | 60.23 |
| TCGA-EE-A2MC | skcm_tcga_pan_can_atlas_2018 | 30 | censored | 0 | 0.68 | 61.51 |
| TCGA-D3-A2JF | skcm_tcga_pan_can_atlas_2018 | 29 | censored | 0 | 0.68 | 62.07 |
| TCGA-GN-A267 | skcm_tcga_pan_can_atlas_2018 | 28 | deceased | 1 | 0.66 | 64.44 |
| TCGA-D3-A2J8 | skcm_tcga_pan_can_atlas_2018 | 27 | deceased | 1 | 0.63 | 65.49 |
| TCGA-HR-A2OH | skcm_tcga_pan_can_atlas_2018 | 26 | deceased | 1 | 0.61 | 65.88 |
| TCGA-D3-A2JO | skcm_tcga_pan_can_atlas_2018 | 25 | censored | 0 | 0.61 | 66.08 |
| TCGA-EB-A5SG | skcm_tcga_pan_can_atlas_2018 | 24 | censored | 0 | 0.61 | 68.25 |
| TCGA-EE-A2MT | skcm_tcga_pan_can_atlas_2018 | 23 | censored | 0 | 0.61 | 71.21 |
| TCGA-D3-A1Q6 | skcm_tcga_pan_can_atlas_2018 | 22 | censored | 0 | 0.61 | 71.80 |
| TCGA-DA-A95X | skcm_tcga_pan_can_atlas_2018 | 21 | censored | 0 | 0.61 | 73.94 |
| TCGA-DA-A1HV | skcm_tcga_pan_can_atlas_2018 | 20 | censored | 0 | 0.61 | 76.57 |
| TCGA-ER-A19A | skcm_tcga_pan_can_atlas_2018 | 19 | censored | 0 | 0.61 | 77.75 |
| TCGA-D3-A2JC | skcm_tcga_pan_can_atlas_2018 | 18 | censored | 0 | 0.61 | 86.76 |
| TCGA-GN-A265 | skcm_tcga_pan_can_atlas_2018 | 17 | censored | 0 | 0.61 | 96.92 |
| TCGA-EE-A2ME | skcm_tcga_pan_can_atlas_2018 | 16 | deceased | 1 | 0.57 | 103.26 |
| TCGA-ER-A1A1 | skcm_tcga_pan_can_atlas_2018 | 15 | censored | 0 | 0.57 | 105.07 |
| TCGA-D3-A8GM | skcm_tcga_pan_can_atlas_2018 | 14 | censored | 0 | 0.57 | 107.14 |
| TCGA-D3-A2JA | skcm_tcga_pan_can_atlas_2018 | 13 | censored | 0 | 0.57 | 115.53 |
| TCGA-EE-A3AB | skcm_tcga_pan_can_atlas_2018 | 12 | censored | 0 | 0.57 | 122.73 |
| TCGA-D3-A1Q7 | skcm_tcga_pan_can_atlas_2018 | 11 | censored | 0 | 0.57 | 133.25 |
| TCGA-EE-A2MR | skcm_tcga_pan_can_atlas_2018 | 10 | censored | 0 | 0.57 | 134.40 |
| TCGA-D3-A5GN | skcm_tcga_pan_can_atlas_2018 | 9 | censored | 0 | 0.57 | 135.75 |
| TCGA-ER-A19W | skcm_tcga_pan_can_atlas_2018 | 8 | deceased | 1 | 0.50 | 148.17 |
| TCGA-ER-A19P | skcm_tcga_pan_can_atlas_2018 | 7 | deceased | 1 | 0.43 | 162.08 |
| TCGA-FR-A44A | skcm_tcga_pan_can_atlas_2018 | 6 | censored | 0 | 0.43 | 174.21 |
| TCGA-D3-A5GR | skcm_tcga_pan_can_atlas_2018 | 5 | censored | 0 | 0.43 | 178.32 |
| TCGA-EE-A3JB | skcm_tcga_pan_can_atlas_2018 | 4 | censored | 0 | 0.43 | 201.80 |
| TCGA-EE-A2MI | skcm_tcga_pan_can_atlas_2018 | 3 | deceased | 1 | 0.28 | 204.66 |
| TCGA-ER-A19G | skcm_tcga_pan_can_atlas_2018 | 2 | censored | 0 | 0.28 | 302.07 |
| TCGA-FS-A1ZC | skcm_tcga_pan_can_atlas_2018 | 1 | deceased | 1 | 0.00 | 357.37 |
|  |  |  |  |  |  |  |
| **(B) cGAS Low** | | | | | | |
| **Case ID** | **Study ID** | **Number at Risk** | **Status** | | **Survival Rate** | **Time (months)** |
| TCGA-GF-A2C7 | skcm_tcga_pan_can_atlas_2018 | 76 | censored | 0 | 1.00 | 0.69 |
| TCGA-EB-A3XE | skcm_tcga_pan_can_atlas_2018 | 75 | censored | 0 | 1.00 | 5.92 |
| TCGA-YD-A89C | skcm_tcga_pan_can_atlas_2018 | 74 | censored | 0 | 1.00 | 6.90 |
| TCGA-QB-A6FS | skcm_tcga_pan_can_atlas_2018 | 73 | censored | 0 | 1.00 | 7.23 |
| TCGA-EB-A553 | skcm_tcga_pan_can_atlas_2018 | 72 | censored | 0 | 1.00 | 7.43 |
| TCGA-EB-A41A | skcm_tcga_pan_can_atlas_2018 | 71 | censored | 0 | 1.00 | 8.61 |
| TCGA-ER-A19T | skcm_tcga_pan_can_atlas_2018 | 70 | deceased | 1 | 0.99 | 8.88 |
| TCGA-FS-A1ZG | skcm_tcga_pan_can_atlas_2018 | 69 | deceased | 1 | 0.97 | 9.70 |
| TCGA-GN-A4U7 | skcm_tcga_pan_can_atlas_2018 | 68 | deceased | 1 | 0.96 | 10.42 |
| TCGA-BF-A5EQ | skcm_tcga_pan_can_atlas_2018 | 67 | censored | 0 | 0.96 | 10.62 |
| TCGA-BF-A5ER | skcm_tcga_pan_can_atlas_2018 | 66 | censored | 0 | 0.96 | 10.75 |
| TCGA-D3-A2JK | skcm_tcga_pan_can_atlas_2018 | 65 | deceased | 1 | 0.94 | 12.10 |
| TCGA-EB-A5SF | skcm_tcga_pan_can_atlas_2018 | 64 | censored | 0 | 0.94 | 12.13 |
| TCGA-DA-A95Z | skcm_tcga_pan_can_atlas_2018 | 63 | censored | 0 | 0.94 | 13.02 |
| TCGA-EB-A5SE | skcm_tcga_pan_can_atlas_2018 | 62 | deceased | 1 | 0.93 | 13.18 |
| TCGA-XV-AB01 | skcm_tcga_pan_can_atlas_2018 | 61 | censored | 0 | 0.93 | 13.25 |
| TCGA-D3-A3ML | skcm_tcga_pan_can_atlas_2018 | 60 | deceased | 1 | 0.91 | 13.87 |
| TCGA-YG-AA3P | skcm_tcga_pan_can_atlas_2018 | 59 | censored | 0 | 0.91 | 14.43 |
| TCGA-EE-A182 | skcm_tcga_pan_can_atlas_2018 | 58 | deceased | 1 | 0.90 | 14.70 |
| TCGA-ER-A19K | skcm_tcga_pan_can_atlas_2018 | 57 | deceased | 1 | 0.88 | 15.42 |
| TCGA-D3-A5GT | skcm_tcga_pan_can_atlas_2018 | 56 | censored | 0 | 0.88 | 16.01 |
| TCGA-BF-A5ES | skcm_tcga_pan_can_atlas_2018 | 55 | censored | 0 | 0.88 | 16.11 |
| TCGA-IH-A3EA | skcm_tcga_pan_can_atlas_2018 | 54 | censored | 0 | 0.88 | 17.23 |
| TCGA-EB-A5KH | skcm_tcga_pan_can_atlas_2018 | 53 | censored | 0 | 0.88 | 20.35 |
| TCGA-DA-A1I0 | skcm_tcga_pan_can_atlas_2018 | 52 | deceased | 1 | 0.86 | 20.38 |
| TCGA-GN-A8LL | skcm_tcga_pan_can_atlas_2018 | 51 | deceased | 1 | 0.85 | 21.37 |
| TCGA-WE-AAA3 | skcm_tcga_pan_can_atlas_2018 | 50 | censored | 0 | 0.85 | 21.40 |
| TCGA-FR-A3R1 | skcm_tcga_pan_can_atlas_2018 | 49 | censored | 0 | 0.85 | 22.52 |
| TCGA-BF-A3DL | skcm_tcga_pan_can_atlas_2018 | 48 | censored | 0 | 0.85 | 25.28 |
| TCGA-EB-A5UM | skcm_tcga_pan_can_atlas_2018 | 47 | censored | 0 | 0.85 | 25.61 |
| TCGA-EE-A29V | skcm_tcga_pan_can_atlas_2018 | 46 | deceased | 1 | 0.83 | 25.87 |
| TCGA-D3-A51T | skcm_tcga_pan_can_atlas_2018 | 45 | censored | 0 | 0.83 | 26.89 |
| TCGA-FS-A1ZZ | skcm_tcga_pan_can_atlas_2018 | 44 | deceased | 1 | 0.81 | 27.02 |
| TCGA-FS-A1ZY | skcm_tcga_pan_can_atlas_2018 | 43 | deceased | 1 | 0.79 | 27.09 |
| TCGA-BF-A1Q0 | skcm_tcga_pan_can_atlas_2018 | 42 | censored | 0 | 0.79 | 27.32 |
| TCGA-D3-A2JE | skcm_tcga_pan_can_atlas_2018 | 41 | deceased | 1 | 0.77 | 27.65 |
| TCGA-FR-A7U8 | skcm_tcga_pan_can_atlas_2018 | 40 | censored | 0 | 0.77 | 27.85 |
| TCGA-ER-A2NB | skcm_tcga_pan_can_atlas_2018 | 39 | deceased | 1 | 0.75 | 28.18 |
| TCGA-FS-A4F5 | skcm_tcga_pan_can_atlas_2018 | 38 | deceased | 1 | 0.73 | 28.73 |
| TCGA-ER-A2NF | skcm_tcga_pan_can_atlas_2018 | 37 | deceased | 1 | 0.71 | 28.83 |
| TCGA-EB-A4OY | skcm_tcga_pan_can_atlas_2018 | 36 | censored | 0 | 0.71 | 32.12 |
| TCGA-D3-A51K | skcm_tcga_pan_can_atlas_2018 | 35 | censored | 0 | 0.71 | 32.94 |
| TCGA-FS-A4F9 | skcm_tcga_pan_can_atlas_2018 | 34 | censored | 0 | 0.71 | 34.03 |
| TCGA-FR-A8YC | skcm_tcga_pan_can_atlas_2018 | 33 | deceased | 1 | 0.69 | 34.82 |
| TCGA-FR-A8YD | skcm_tcga_pan_can_atlas_2018 | 32 | deceased | 1 | 0.67 | 36.26 |
| TCGA-DA-A95W | skcm_tcga_pan_can_atlas_2018 | 31 | censored | 0 | 0.67 | 37.35 |
| TCGA-EB-A3XD | skcm_tcga_pan_can_atlas_2018 | 30 | censored | 0 | 0.67 | 38.14 |
| TCGA-GN-A4U4 | skcm_tcga_pan_can_atlas_2018 | 29 | censored | 0 | 0.67 | 39.35 |
| TCGA-ER-A194 | skcm_tcga_pan_can_atlas_2018 | 28 | deceased | 1 | 0.64 | 44.51 |
| TCGA-ER-A3EV | skcm_tcga_pan_can_atlas_2018 | 27 | deceased | 1 | 0.62 | 46.98 |
| TCGA-FS-A1ZJ | skcm_tcga_pan_can_atlas_2018 | 26 | deceased | 1 | 0.60 | 47.37 |
| TCGA-EB-A5SH | skcm_tcga_pan_can_atlas_2018 | 25 | censored | 0 | 0.60 | 54.02 |
| TCGA-FS-A4FC | skcm_tcga_pan_can_atlas_2018 | 24 | deceased | 1 | 0.57 | 54.41 |
| TCGA-D3-A8GI | skcm_tcga_pan_can_atlas_2018 | 23 | deceased | 1 | 0.55 | 58.52 |
| TCGA-EE-A29S | skcm_tcga_pan_can_atlas_2018 | 22 | deceased | 1 | 0.52 | 61.28 |
| TCGA-GN-A268 | skcm_tcga_pan_can_atlas_2018 | 21 | deceased | 1 | 0.50 | 62.79 |
| TCGA-FS-A1ZP | skcm_tcga_pan_can_atlas_2018 | 20 | deceased | 1 | 0.47 | 74.73 |
| TCGA-D9-A6EC | skcm_tcga_pan_can_atlas_2018 | 19 | censored | 0 | 0.47 | 77.56 |
| TCGA-FS-A4F0 | skcm_tcga_pan_can_atlas_2018 | 18 | censored | 0 | 0.47 | 77.82 |
| TCGA-D3-A8GC | skcm_tcga_pan_can_atlas_2018 | 17 | deceased | 1 | 0.44 | 79.59 |
| TCGA-EE-A29B | skcm_tcga_pan_can_atlas_2018 | 16 | deceased | 1 | 0.42 | 85.08 |
| TCGA-D3-A8GL | skcm_tcga_pan_can_atlas_2018 | 15 | deceased | 1 | 0.39 | 89.13 |
| TCGA-D3-A1QA | skcm_tcga_pan_can_atlas_2018 | 14 | censored | 0 | 0.39 | 90.90 |
| TCGA-EE-A180 | skcm_tcga_pan_can_atlas_2018 | 13 | deceased | 1 | 0.36 | 94.98 |
| TCGA-W3-AA21 | skcm_tcga_pan_can_atlas_2018 | 12 | deceased | 1 | 0.33 | 105.04 |
| TCGA-W3-AA1R | skcm_tcga_pan_can_atlas_2018 | 11 | deceased | 1 | 0.30 | 111.09 |
| TCGA-D3-A2JG | skcm_tcga_pan_can_atlas_2018 | 10 | deceased | 1 | 0.27 | 113.52 |
| TCGA-EE-A3J4 | skcm_tcga_pan_can_atlas_2018 | 9 | deceased | 1 | 0.24 | 127.20 |
| TCGA-D3-A8GP | skcm_tcga_pan_can_atlas_2018 | 8 | censored | 0 | 0.24 | 152.74 |
| TCGA-EE-A3JI | skcm_tcga_pan_can_atlas_2018 | 7 | deceased | 1 | 0.21 | 152.81 |
| TCGA-EE-A2MS | skcm_tcga_pan_can_atlas_2018 | 6 | censored | 0 | 0.21 | 162.47 |
| TCGA-EE-A2MM | skcm_tcga_pan_can_atlas_2018 | 5 | deceased | 1 | 0.16 | 167.90 |
| TCGA-EE-A20H | skcm_tcga_pan_can_atlas_2018 | 4 | deceased | 1 | 0.12 | 168.26 |
| TCGA-DA-A3F5 | skcm_tcga_pan_can_atlas_2018 | 3 | deceased | 1 | 0.08 | 225.96 |
| TCGA-FS-A1YY | skcm_tcga_pan_can_atlas_2018 | 2 | deceased | 1 | 0.04 | 228.59 |
| TCGA-ER-A3ES | skcm_tcga_pan_can_atlas_2018 | 1 | deceased | 1 | 0.00 | 247.03 |

**Supplementary Table 6. Expression values of *CCL5*, *CXCL10*, *CD8A*, *CD8B*, *NCR1*, and *NCR3* of the melanoma patients expressing high and low *cGAS***

|  |  | **mRNA Expression,**  **RSEM (Batch normalized from Illumina HiSeq_RNASeqV2) (log2)** | | | | | |
| --- | --- | --- | --- | --- | --- | --- | --- |
| **Sample Id** | **Group** | ***CCL5*** | ***CXCL10*** | ***CD8A*** | ***CD8B*** | ***NCR1*** | ***NCR3*** |
| TCGA-D3-A1Q6-06 | (A) cGAS High | 7.79 | 8.59 | 6.65 | 4.10 | 0.94 | 2.23 |
| TCGA-D3-A1Q7-06 | (A) cGAS High | 12.31 | 12.77 | 11.57 | 9.55 | 3.69 | 4.56 |
| TCGA-D3-A1Q8-06 | (A) cGAS High | 7.70 | 6.27 | 6.65 | 4.93 | 1.01 | 3.72 |
| TCGA-D3-A2J8-06 | (A) cGAS High | 12.51 | 11.87 | 10.95 | 9.15 | 2.34 | 5.32 |
| TCGA-D3-A2J9-06 | (A) cGAS High | 13.39 | 11.78 | 12.60 | 10.85 | 4.07 | 6.97 |
| TCGA-D3-A2JA-06 | (A) cGAS High | 9.97 | 10.76 | 8.49 | 6.16 | 1.89 | 3.04 |
| TCGA-D3-A2JC-06 | (A) cGAS High | 10.96 | 9.20 | 9.49 | 7.33 | 1.59 | 3.88 |
| TCGA-D3-A2JF-06 | (A) cGAS High | 11.27 | 11.49 | 9.49 | 8.00 | 1.82 | 5.62 |
| TCGA-D3-A2JO-06 | (A) cGAS High | 12.40 | 10.65 | 10.63 | 8.97 | 2.58 | 4.90 |
| TCGA-D3-A3C8-06 | (A) cGAS High | 14.47 | 13.70 | 13.45 | 11.54 | 2.42 | 5.18 |
| TCGA-D3-A3CE-06 | (A) cGAS High | 11.03 | 10.77 | 10.46 | 8.16 | 4.41 | 4.95 |
| TCGA-D3-A51F-06 | (A) cGAS High | 14.55 | 11.39 | 13.15 | 10.95 | 5.35 | 5.89 |
| TCGA-D3-A51G-06 | (A) cGAS High | 9.50 | 8.85 | 8.76 | 6.53 | 1.96 | 1.29 |
| TCGA-D3-A51H-06 | (A) cGAS High | 11.45 | 9.74 | 9.62 | 7.67 | 2.61 | 8.23 |
| TCGA-D3-A51N-06 | (A) cGAS High | 10.30 | 10.74 | 7.76 | 5.02 | 0.00 | 4.35 |
| TCGA-D3-A5GN-06 | (A) cGAS High | 10.20 | 10.38 | 9.21 | 7.09 | 2.01 | 3.41 |
| TCGA-D3-A5GR-06 | (A) cGAS High | 9.92 | 9.49 | 8.59 | 6.89 | 2.42 | 6.88 |
| TCGA-D3-A8GM-06 | (A) cGAS High | 9.74 | 8.52 | 8.53 | 6.52 | 1.60 | 2.89 |
| TCGA-DA-A1HV-06 | (A) cGAS High | 11.40 | 11.37 | 10.29 | 8.52 | 2.49 | 4.41 |
| TCGA-DA-A1IB-06 | (A) cGAS High | 12.61 | 10.89 | 11.29 | 9.31 | 3.53 | 7.83 |
| TCGA-DA-A3F8-06 | (A) cGAS High | 13.17 | 12.60 | 12.34 | 10.51 | 4.17 | 4.55 |
| TCGA-DA-A95X-06 | (A) cGAS High | 8.84 | 9.12 | 7.79 | 5.54 | 0.41 | 2.30 |
| TCGA-EB-A5SG-06 | (A) cGAS High | 13.74 | 12.89 | 12.45 | 10.68 | 3.77 | 5.75 |
| TCGA-EB-A5VV-06 | (A) cGAS High | 12.60 | 10.58 | 10.76 | 9.37 | 2.50 | 8.71 |
| TCGA-EB-A6QY-01 | (A) cGAS High | 7.25 | 6.41 | 5.94 | 3.67 | 0.46 | 1.09 |
| TCGA-EE-A17Y-06 | (A) cGAS High | 6.63 | 5.35 | 5.13 | 1.96 | 0.45 | 0.79 |
| TCGA-EE-A20C-06 | (A) cGAS High | 4.67 | 3.87 | 3.11 | 0.73 | 0.00 | 0.00 |
| TCGA-EE-A29N-06 | (A) cGAS High | 10.36 | 9.72 | 8.98 | 7.10 | 0.87 | 5.88 |
| TCGA-EE-A2GK-06 | (A) cGAS High | 11.78 | 8.26 | 10.47 | 9.05 | 2.61 | 9.27 |
| TCGA-EE-A2M5-06 | (A) cGAS High | 10.08 | 9.27 | 9.36 | 7.14 | 1.46 | 2.05 |
| TCGA-EE-A2M8-06 | (A) cGAS High | 13.66 | 12.20 | 12.51 | 10.71 | 5.39 | 6.05 |
| TCGA-EE-A2MC-06 | (A) cGAS High | 12.02 | 11.08 | 10.82 | 8.95 | 1.77 | 4.70 |
| TCGA-EE-A2ME-06 | (A) cGAS High | 12.50 | 11.97 | 11.82 | 10.36 | 3.79 | 6.48 |
| TCGA-EE-A2MI-06 | (A) cGAS High | 9.79 | 9.29 | 9.29 | 6.74 | 1.32 | 2.97 |
| TCGA-EE-A2MR-06 | (A) cGAS High | 11.01 | 9.91 | 10.54 | 9.04 | 3.12 | 6.34 |
| TCGA-EE-A2MT-06 | (A) cGAS High | 8.46 | 7.09 | 6.82 | 4.61 | 0.00 | 1.61 |
| TCGA-EE-A3AB-06 | (A) cGAS High | 9.05 | 6.90 | 7.57 | 5.20 | 0.63 | 4.75 |
| TCGA-EE-A3AG-06 | (A) cGAS High | 6.64 | 5.54 | 5.79 | 8.37 | 0.36 | 1.84 |
| TCGA-EE-A3JB-06 | (A) cGAS High | 8.89 | 9.71 | 6.61 | 3.92 | 0.00 | 3.60 |
| TCGA-EE-A3JD-06 | (A) cGAS High | 11.38 | 11.27 | 10.24 | 8.89 | 0.00 | 6.02 |
| TCGA-EE-A3JE-06 | (A) cGAS High | 13.25 | 13.38 | 12.29 | 9.98 | 4.75 | 5.02 |
| TCGA-ER-A195-06 | (A) cGAS High | 10.70 | 9.61 | 10.21 | 8.04 | 3.79 | 4.64 |
| TCGA-ER-A198-06 | (A) cGAS High | 9.87 | 9.51 | 6.94 | 4.33 | 0.88 | 3.74 |
| TCGA-ER-A19A-06 | (A) cGAS High | 12.25 | 12.28 | 10.94 | 8.76 | 3.42 | 4.82 |
| TCGA-ER-A19G-06 | (A) cGAS High | 11.06 | 12.53 | 8.50 | 6.16 | 3.62 | 4.47 |
| TCGA-ER-A19P-06 | (A) cGAS High | 12.87 | 11.80 | 12.13 | 10.56 | 4.75 | 6.85 |
| TCGA-ER-A19Q-06 | (A) cGAS High | 10.33 | 9.74 | 8.88 | 7.17 | 0.96 | 3.24 |
| TCGA-ER-A19S-06 | (A) cGAS High | 13.22 | 12.08 | 12.43 | 10.90 | 3.53 | 4.91 |
| TCGA-ER-A19W-06 | (A) cGAS High | 12.59 | 13.09 | 11.65 | 9.92 | 3.65 | 5.78 |
| TCGA-ER-A1A1-06 | (A) cGAS High | 10.02 | 8.14 | 9.18 | 7.50 | 3.46 | 8.44 |
| TCGA-ER-A2NH-06 | (A) cGAS High | 13.10 | 12.39 | 12.21 | 10.48 | 4.64 | 4.41 |
| TCGA-FR-A44A-06 | (A) cGAS High | 13.30 | 12.83 | 11.77 | 9.37 | 4.16 | 5.36 |
| TCGA-FR-A7U9-06 | (A) cGAS High | 10.82 | 10.24 | 8.11 | 6.48 | 0.98 | 3.96 |
| TCGA-FR-A7UA-06 | (A) cGAS High | 14.07 | 13.48 | 13.41 | 11.48 | 4.57 | 6.26 |
| TCGA-FS-A1Z4-06 | (A) cGAS High | 10.51 | 10.24 | 8.70 | 6.79 | 2.63 | 6.25 |
| TCGA-FS-A1ZC-06 | (A) cGAS High | 4.34 | 3.83 | 4.18 | 2.75 | 0.38 | 1.77 |
| TCGA-FS-A1ZD-06 | (A) cGAS High | 5.49 | 4.06 | 9.70 | 1.61 | 2.97 | 3.14 |
| TCGA-FS-A1ZW-06 | (A) cGAS High | 10.21 | 11.47 | 9.94 | 7.84 | 2.27 | 3.28 |
| TCGA-GF-A3OT-06 | (A) cGAS High | 10.10 | 9.91 | 8.48 | 6.50 | 2.11 | 2.32 |
| TCGA-GF-A4EO-06 | (A) cGAS High | 12.65 | 11.84 | 11.57 | 10.16 | 3.02 | 5.87 |
| TCGA-GF-A769-01 | (A) cGAS High | 6.24 | 5.51 | 4.47 | 2.85 | 0.45 | 0.79 |
| TCGA-GN-A265-06 | (A) cGAS High | 11.68 | 11.45 | 9.27 | 7.35 | 2.30 | 4.68 |
| TCGA-GN-A266-06 | (A) cGAS High | 10.70 | 11.74 | 9.73 | 7.03 | 2.95 | 3.64 |
| TCGA-GN-A267-06 | (A) cGAS High | 10.81 | 9.57 | 9.72 | 8.23 | 0.70 | 2.24 |
| TCGA-GN-A26C-01 | (A) cGAS High | 12.70 | 10.98 | 11.11 | 8.75 | 2.01 | 4.89 |
| TCGA-HR-A2OH-06 | (A) cGAS High | 13.08 | 13.02 | 12.54 | 10.59 | 5.11 | 6.29 |
| TCGA-W3-AA1V-06 | (A) cGAS High | 10.41 | 8.73 | 7.00 | 4.95 | 2.46 | 3.02 |
| TCGA-YD-A9TA-06 | (A) cGAS High | 9.91 | 9.16 | 8.35 | 5.82 | 2.52 | 3.95 |
| TCGA-BF-A1Q0-01 | (B) cGAS Low | 9.19 | 8.92 | 7.73 | 6.05 | 0.77 | 3.39 |
| TCGA-BF-A3DL-01 | (B) cGAS Low | 8.12 | 4.06 | 5.54 | 3.78 | 0.58 | 0.58 |
| TCGA-BF-A5EQ-01 | (B) cGAS Low | 10.12 | 8.91 | 8.97 | 7.25 | 2.74 | 3.68 |
| TCGA-BF-A5ER-01 | (B) cGAS Low | 7.87 | 3.03 | 2.94 | 2.62 | 0.00 | 0.60 |
| TCGA-BF-A5ES-01 | (B) cGAS Low | 9.56 | 7.79 | 6.85 | 4.98 | 0.00 | 1.11 |
| TCGA-D3-A1QA-06 | (B) cGAS Low | 8.67 | 8.54 | 7.39 | 5.54 | 0.58 | 1.90 |
| TCGA-D3-A2JE-06 | (B) cGAS Low | 6.56 | 6.20 | 4.15 | 2.25 | 0.00 | 2.69 |
| TCGA-D3-A2JG-06 | (B) cGAS Low | 8.16 | 6.43 | 6.23 | 4.41 | 0.00 | 0.81 |
| TCGA-D3-A2JK-06 | (B) cGAS Low | 9.35 | 4.60 | 5.17 | 0.00 | 0.00 | 3.13 |
| TCGA-D3-A3C1-06 | (B) cGAS Low | 9.23 | 3.52 | 7.17 | 5.84 | 0.85 | 3.24 |
| TCGA-D3-A3ML-06 | (B) cGAS Low | 5.18 | 4.82 | 3.73 | 1.49 | 0.00 | 0.68 |
| TCGA-D3-A51K-06 | (B) cGAS Low | 10.59 | 7.62 | 8.50 | 7.26 | 0.00 | 3.14 |
| TCGA-D3-A51T-06 | (B) cGAS Low | 9.76 | 8.98 | 7.56 | 5.50 | 0.59 | 3.81 |
| TCGA-D3-A5GT-01 | (B) cGAS Low | 8.66 | 3.85 | 2.95 | 0.00 | 3.77 | 0.80 |
| TCGA-D3-A8GC-06 | (B) cGAS Low | 7.65 | 6.39 | 6.26 | 4.34 | 0.45 | 2.23 |
| TCGA-D3-A8GI-06 | (B) cGAS Low | 7.06 | 4.84 | 4.30 | 1.48 | 1.48 | 1.88 |
| TCGA-D3-A8GL-06 | (B) cGAS Low | 4.77 | 3.82 | 1.95 | 0.50 | 0.00 | 0.00 |
| TCGA-D3-A8GP-06 | (B) cGAS Low | 7.87 | 5.44 | 6.73 | 4.79 | 1.06 | 1.40 |
| TCGA-D3-A8GR-06 | (B) cGAS Low | 7.77 | 4.72 | 4.44 | 3.24 | 0.00 | 0.96 |
| TCGA-D9-A6EC-06 | (B) cGAS Low | 8.62 | 8.87 | 4.27 | 2.69 | 0.72 | 0.97 |
| TCGA-DA-A1I0-06 | (B) cGAS Low | 8.92 | 3.59 | 7.41 | 6.02 | 2.02 | 5.57 |
| TCGA-DA-A3F5-06 | (B) cGAS Low | 7.97 | 4.29 | 6.52 | 3.89 | 0.48 | 1.37 |
| TCGA-DA-A95W-06 | (B) cGAS Low | 9.97 | 8.09 | 8.44 | 6.52 | 0.59 | 1.32 |
| TCGA-DA-A95Z-06 | (B) cGAS Low | 8.30 | 7.78 | 6.19 | 4.36 | 0.00 | 1.66 |
| TCGA-EB-A3XD-01 | (B) cGAS Low | 10.86 | 7.41 | 8.95 | 7.34 | 1.52 | 5.46 |
| TCGA-EB-A3XE-01 | (B) cGAS Low | 10.25 | 8.72 | 8.10 | 6.90 | 0.00 | 3.06 |
| TCGA-EB-A41A-01 | (B) cGAS Low | 7.79 | 7.84 | 5.77 | 4.17 | 0.00 | 1.73 |
| TCGA-EB-A4OY-01 | (B) cGAS Low | 10.59 | 5.37 | 8.16 | 6.67 | 0.00 | 2.84 |
| TCGA-EB-A553-01 | (B) cGAS Low | 10.58 | 6.41 | 7.71 | 5.60 | 0.99 | 0.00 |
| TCGA-EB-A5KH-06 | (B) cGAS Low | 7.45 | 3.86 | 4.74 | 2.02 | 0.00 | 2.40 |
| TCGA-EB-A5SE-01 | (B) cGAS Low | 7.85 | 7.58 | 6.33 | 4.23 | 0.00 | 0.79 |
| TCGA-EB-A5SF-01 | (B) cGAS Low | 7.12 | 2.33 | 3.95 | 2.08 | 0.00 | 2.33 |
| TCGA-EB-A5SH-06 | (B) cGAS Low | 8.77 | 6.52 | 7.06 | 5.70 | 3.21 | 4.63 |
| TCGA-EB-A5UM-01 | (B) cGAS Low | 10.85 | 8.45 | 9.70 | 7.49 | 0.72 | 5.57 |
| TCGA-EE-A180-06 | (B) cGAS Low | 7.98 | 8.62 | 6.48 | 4.22 | 0.00 | 0.68 |
| TCGA-EE-A182-06 | (B) cGAS Low | 10.29 | 4.19 | 6.99 | 6.05 | 0.00 | 3.90 |
| TCGA-EE-A20H-06 | (B) cGAS Low | 6.81 | 6.77 | 5.99 | 3.46 | 0.35 | 1.81 |
| TCGA-EE-A29B-06 | (B) cGAS Low | 8.54 | 5.50 | 5.91 | 3.34 | 0.00 | 0.90 |
| TCGA-EE-A29L-06 | (B) cGAS Low | 4.83 | 3.82 | 2.67 | 1.14 | 0.00 | 0.92 |
| TCGA-EE-A29S-06 | (B) cGAS Low | 9.73 | 7.49 | 8.65 | 6.60 | 0.38 | 3.53 |
| TCGA-EE-A29V-06 | (B) cGAS Low | 6.57 | 2.55 | 3.76 | 2.48 | 0.65 | 1.44 |
| TCGA-EE-A2MM-06 | (B) cGAS Low | 7.75 | 10.00 | 6.31 | 4.45 | 0.38 | 2.67 |
| TCGA-EE-A2MS-06 | (B) cGAS Low | 10.35 | 7.97 | 8.83 | 6.33 | 1.67 | 3.04 |
| TCGA-EE-A3J4-06 | (B) cGAS Low | 8.80 | 10.29 | 6.84 | 4.98 | 0.43 | 1.02 |
| TCGA-EE-A3JI-06 | (B) cGAS Low | 7.47 | 8.50 | 6.15 | 4.39 | 0.00 | 2.25 |
| TCGA-ER-A194-01 | (B) cGAS Low | 11.92 | 7.33 | 9.12 | 7.12 | 2.70 | 2.83 |
| TCGA-ER-A19K-01 | (B) cGAS Low | 9.53 | 9.03 | 7.26 | 5.30 | 1.01 | 2.34 |
| TCGA-ER-A19T-01 | (B) cGAS Low | 4.87 | 3.44 | 4.38 | 0.54 | 0.54 | 0.00 |
| TCGA-ER-A19T-06 | (B) cGAS Low | 9.53 | 7.88 | 7.53 | 4.92 | 0.45 | 3.24 |
| TCGA-ER-A2NB-01 | (B) cGAS Low | 12.45 | 6.41 | 9.17 | 8.21 | 1.84 | 3.65 |
| TCGA-ER-A2NF-01 | (B) cGAS Low | 10.59 | 9.00 | 9.40 | 7.84 | 2.20 | 4.47 |
| TCGA-ER-A2NF-06 | (B) cGAS Low | 10.35 | 8.07 | 9.30 | 7.79 | 2.46 | 3.33 |
| TCGA-ER-A3ES-06 | (B) cGAS Low | 5.06 | 3.19 | 3.77 | 1.49 | 0.00 | 0.00 |
| TCGA-ER-A3EV-06 | (B) cGAS Low | 10.42 | 7.23 | 9.03 | 7.27 | 1.71 | 3.34 |
| TCGA-FR-A3R1-01 | (B) cGAS Low | 9.94 | 10.39 | 8.66 | 6.32 | 0.41 | 1.58 |
| TCGA-FR-A7U8-06 | (B) cGAS Low | 6.03 | 5.62 | 4.87 | 2.16 | 0.00 | 1.58 |
| TCGA-FR-A8YC-06 | (B) cGAS Low | 9.47 | 8.00 | 9.08 | 6.56 | 2.16 | 1.11 |
| TCGA-FR-A8YD-06 | (B) cGAS Low | 8.25 | 4.44 | 6.55 | 4.27 | 0.00 | 3.30 |
| TCGA-FS-A1YY-06 | (B) cGAS Low | 6.55 | 3.59 | 3.92 | 3.03 | 0.82 | 0.60 |
| TCGA-FS-A1ZG-06 | (B) cGAS Low | 6.91 | 5.30 | 4.23 | 2.19 | 0.63 | 0.63 |
| TCGA-FS-A1ZJ-06 | (B) cGAS Low | 8.83 | 8.03 | 8.17 | 5.57 | 1.20 | 1.56 |
| TCGA-FS-A1ZP-06 | (B) cGAS Low | 8.77 | 7.50 | 7.72 | 6.21 | 0.82 | 3.40 |
| TCGA-FS-A1ZY-06 | (B) cGAS Low | 5.90 | 4.31 | 3.91 | 1.84 | 0.00 | 0.00 |
| TCGA-FS-A1ZZ-06 | (B) cGAS Low | 8.31 | 7.39 | 4.67 | 3.12 | 0.00 | 0.34 |
| TCGA-FS-A4F0-06 | (B) cGAS Low | 5.88 | 6.04 | 3.21 | 1.61 | 0.00 | 0.67 |
| TCGA-FS-A4F5-06 | (B) cGAS Low | 6.83 | 9.27 | 4.57 | 2.15 | 0.43 | 0.76 |
| TCGA-FS-A4F9-06 | (B) cGAS Low | 6.04 | 5.30 | 5.17 | 3.18 | 0.82 | 0.00 |
| TCGA-FS-A4FC-06 | (B) cGAS Low | 8.78 | 8.56 | 7.22 | 5.25 | 0.00 | 3.41 |
| TCGA-GF-A2C7-01 | (B) cGAS Low | 7.49 | 2.06 | 3.39 | 0.40 | 0.00 | 2.06 |
| TCGA-GN-A268-06 | (B) cGAS Low | 6.68 | 6.01 | 5.53 | 3.15 | 0.00 | 0.70 |
| TCGA-GN-A4U4-06 | (B) cGAS Low | 7.16 | 7.03 | 6.45 | 4.78 | 0.44 | 1.93 |
| TCGA-GN-A4U7-06 | (B) cGAS Low | 5.96 | 2.72 | 2.92 | 1.60 | 0.00 | 2.01 |
| TCGA-GN-A8LL-06 | (B) cGAS Low | 8.69 | 3.25 | 6.74 | 5.03 | 2.99 | 1.73 |
| TCGA-IH-A3EA-01 | (B) cGAS Low | 9.19 | 4.25 | 5.32 | 4.17 | 0.00 | 0.00 |
| TCGA-QB-A6FS-06 | (B) cGAS Low | 11.88 | 7.70 | 9.97 | 8.63 | 2.33 | 5.98 |
| TCGA-RP-A695-06 | (B) cGAS Low | 9.45 | 5.63 | 8.32 | 6.24 | 0.51 | 3.12 |
| TCGA-W3-AA1R-06 | (B) cGAS Low | 9.73 | 8.99 | 8.19 | 6.45 | 1.05 | 1.05 |
| TCGA-W3-AA21-06 | (B) cGAS Low | 8.71 | 8.32 | 7.16 | 5.49 | 0.00 | 1.36 |
| TCGA-WE-AAA3-06 | (B) cGAS Low | 10.88 | 8.80 | 9.87 | 7.56 | 2.36 | 2.23 |
| TCGA-XV-AB01-06 | (B) cGAS Low | 10.38 | 5.88 | 7.82 | 6.21 | 1.25 | 3.88 |
| TCGA-YD-A89C-06 | (B) cGAS Low | 5.58 | 4.45 | 3.85 | 2.82 | 0.00 | 0.90 |
| TCGA-YG-AA3P-06 | (B) cGAS Low | 6.66 | 8.60 | 5.94 | 2.53 | 1.05 | 0.62 |

**References for Supplementary Tables**

1 Kabsch W. XDS. *Acta Crystallogr D Biol Crystallogr*. 2010;**66**(Pt 2):125–132.

2 McCoy AJ, Grosse-Kunstleve RW, Adams PD, Winn MD, Storoni LC, Read RJ. Phaser crystallographic software. *J Appl Crystallogr*. 2007;**40**(4):658–674.

3 Berman HM, Westbrook J, Feng Z, Gilliland G, Bhat TN, Weissig H, et al. The Protein Data Bank. *Nucleic Acids Res*. 2000;**28**(1):235–242.

4 Trésaugues L, Welin M, Arrowsmith, C.H. Berglund H, Bountra C, Collins R, Edwards AM, et al. Crystal structure of human PIK3C3 in complex with 3-[4-(4-Morpholinyl)thieno[3,2-d]pyrimidin-2-yl]-phenol. 2010. https://doi.org/10.2210/pdb3LS8/pdb.

5 Emsley P, Lohkamp B, Scott WG, Cowtan K. Features and development of Coot. *Acta Crystallogr D Biol Crystallogr*. 2010;**66**(Pt 4):486–501.

6 Murshudov GN, Vagin AA, Dodson EJ, IUCr. Refinement of Macromolecular Structures by the Maximum-Likelihood Method. *Acta Crystallogr Sect D Biol Crystallogr*. 1997;**53**(3):240–255.

7 Williams CJ, Headd JJ, Moriarty NW, Prisant MG, Videau LL, Deis LN, et al. MolProbity: More and better reference data for improved all-atom structure validation. *Protein Sci*. 2018;**27**(1):293–315.

8 Karplus PA, Diederichs K. Linking crystallographic model and data quality. *Science*. 2012;**336**(6084):1030–1033.

9 Diederichs K, Karplus PA. Improved R-factors for diffraction data analysis in macromolecular crystallography. *Nat Struct Biol*. 1997;**4**(4):269–275.

10 Merid SK, Goranskaya D, Alexeyenko A. Distinguishing between driver and passenger mutations in individual cancer genomes by network enrichment analysis. *BMC Bioinformatics*. 2014;**15**(1):308.

11 Jeggari A, Alexeyenko A. NEArender: An R package for functional interpretation of “omics” data via network enrichment analysis. *BMC Bioinformatics*. 2017;**18**:14–16.

12 Zemek RM, De Jong E, Chin WL, Schuster IS, Fear VS, Casey TH, et al. Sensitization to immune checkpoint blockade through activation of a STAT1/NK axis in the tumor microenvironment. *Sci Transl Med*. 2019;**11**(501):7816.

13 Arensman MD, Yang XS, Zhong W, Bisulco S, Upeslacis E, Rosfjord EC, et al. Anti-tumor immunity influences cancer cell reliance upon ATG7. *Oncoimmunology*. 2020;**9**(1).

14 Poillet-Perez L, Sharp DW, Yang Y, Laddha S V., Ibrahim M, Bommareddy PK, et al. Autophagy promotes growth of tumors with high mutational burden by inhibiting a T-cell immune response. *Nat Cancer*. 2020;**1**(9):923–934.

15 Prabakaran T, Bodda C, Krapp C, Zhang B, Christensen MH, Sun C, et al. Attenuation of cGAS‐STING signaling is mediated by a p62/SQSTM1‐dependent autophagy pathway activated by TBK1. *EMBO J*. 2018;**37**(8):e97858.

16 Wei H, Wei S, Gan B, Peng X, Zou W, Guan JL. Suppression of autophagy by FIP200 deletion inhibits mammary tumorigenesis. *Genes Dev*. 2011;**25**(14):1510–1527.

17 Gonugunta VK, Sakai T, Pokatayev V, Yang K, Wu J, Dobbs N, et al. Trafficking-Mediated STING Degradation Requires Sorting to Acidified Endolysosomes and Can Be Targeted to Enhance Anti-tumor Response. *Cell Rep*. 2017;**21**(11):3234–3242.

18 Noman MZ, Parpal S, Van Moer K, Xiao M, Yu Y, Viklund J, et al. Inhibition of Vps34 reprograms cold into hot inflamed tumors and improves anti–PD-1/PD-L1 immunotherapy. *Sci Adv*. 2020;**6**(18):eaax7881.

19 Lawson KA, Sousa CM, Zhang X, Kim E, Akthar R, Caumanns JJ, et al. Functional genomic landscape of cancer-intrinsic evasion of killing by T cells. *Nature*. 2020;**586**(7827):120–126.
